# Cysteine sulfenylation contributes to liver fibrosis via the regulation of EphB2-mediated signaling

**DOI:** 10.1038/s41419-024-06997-9

**Published:** 2024-08-20

**Authors:** Yueqing Han, Qi Gao, Yating Xu, Ke Chen, Rongxin Li, Weiran Guo, Shuzhen Wang

**Affiliations:** School of Life Science and Technology, China Pharmaceutical University, Nanjing, China

**Keywords:** Liver fibrosis, Kinases, Post-translational modifications

## Abstract

Sulfenylation is a reversible oxidative posttranslational modification (PTM) of proteins on cysteine residues. Despite the dissection of various biological functions of cysteine sulfenylation, its roles in hepatic fibrosis remain elusive. Here, we report that EphB2, a receptor tyrosine kinase previously implicated in liver fibrosis, is regulated by cysteine sulfenylation during the fibrotic progression of liver. Specifically, EphB2 is sulfenylated at the residues of Cys636 and Cys862 in activated hepatic stellate cells (HSCs), leading to the elevation of tyrosine kinase activity and protein stability of EphB2 and stronger interactions with focal adhesion kinase for the activation of downstream mitogen-activated protein kinase signaling. The inhibitions of both EphB2 kinase activity and cysteine sulfenylation by idebenone (IDE), a marketed drug with potent antioxidant activity, can markedly suppress the activation of HSCs and ameliorate hepatic injury in two well-recognized mouse models of liver fibrosis. Collectively, this study reveals cysteine sulfenylation as a new type of PTM for EphB2 and sheds a light on the therapeutic potential of IDE for the treatment of liver fibrosis.

## Introduction

Liver fibrosis is a dynamic wound healing process due to various chronic liver injuries, such as, hepatitis virus infection, autoimmune hepatitis, alcoholic liver diseases, and nonalcoholic fatty liver disease (NAFLD, currently named as metabolic dysfunction-associated steatotic liver disease, MASLD) with or without metabolic associated steatohepatitis (MASH) [1]. If left untreated, hepatic fibrosis can progress into liver cirrhosis, hepatocellular dysfunction and hepatic insufficiency, and even hepatocellular carcinoma (HCC) [2]. Persistent activation and phenotypic transformation of hepatic stellate cells (HSCs), the main producer of extracelluar matrix (ECM), into myofibroblasts has been considered as a central contributors of fibrogenesis [3]. Although the incidence and prevalence are increasing, currently there are no approved effective antifibrotic drugs available on the market, emphasizing an urgent clinical need for novel and specific antifibrotic therapies [4].

The hyperactive receptor tyrosine kinases (RTKs) and related pathways have been implicated in various fibrotic diseases [5–8]. Recently, new insights have been gained on the involvement of the largest family of RTKs - Eph receptors and their ligands Ephrins in tissue fibrosis, making them valuable and attractive targets in fibrotic diseases [9, 10]. For example, EphA3 expression is significantly increased in idiopathic pulmonary fibrosis and antibody-directed killing of EphA3^+^ cells ameliorated pulmonary fibrosis in humanized immunodeficient mice [11]. Ephrin-B2 and its receptors EphB3 and EphB4 can promote fibrosis of multiple organs and blockade of Ephrin-B2 signaling ameliorates or prevents cardiac and lung fibrosis in mice [12, 13]. The profibrogenic roles of EphB2 in liver fibrosis and MASH have been recognized in several different murine models [14–18]. Previously, we reported the transcriptional regulation of EphB2 expression by miR-451 and miR-185 in HSCs [17]. However, it remains not fully explored regarding the regulation of posttranslational modifications (PTMs) other than phosphorylation on the functions and activities of EphB2 during progression of liver fibrosis.

Oxidative imbalance between reactive oxygen species (ROS) production and ROS elimination plays a key role in the pathogenesis of almost all chronic liver diseases [19, 20]. Excessive ROS can target specific proteins via different oxidative PTMs, modulate their biological effects and lead to oxidative damage [21]. Accumulating research findings demonstrate that RTKs are susceptible to reversible oxidative PTMs, such as, sulfenylation, which can alter their kinase activities and contribute to specific redox-regulated signaling events [22, 23]. Protein sulfenylation refers to the oxidation of cysteine residues within target proteins to produce S-sulfenic acid (-SOH) [24], epidermal growth factor receptor (EGFR) and Src are two well-characterized kinase targets activated by sulfenylation [25–28]. Although oxidative PTMs of several ECM proteins have been reported [29], the oxidation of RTKs is still comparatively less well-studied in liver fibrosis.

Considering that a better understanding of EphB2 PTMs may facilitate future researches on novel therapies for liver fibrosis and other EphB2-related disorders, in the present study we investigated the sulfenylation of EphB2 within HSCs during liver fibrosis and provided substantial evidence for the regulatory functions of sulfenylation on EphB2 kinase activity, protein stability, protein-protein interaction and downstream signaling. In addition, we report Idebenone (IDE) as EphB2 tyrosine kinase inhibitor, demonstrate its potent protective effects on liver injury, and propose its potential repurposing for the treatment of liver fibrosis.

## Methods

### Animal

Male C57BL/6 J mice (6–8 weeks, 18–22 g) were purchased from SPF Biotechnology Co., Ltd (Beijing, China), and raised in pathogen-free, constant temperature, 40–60% humidity, 12-hour light/dark cycle, dry feedstuff, and water *ad libitum*. All mice received human care and animal experiments complied with health guidelines and approved by Animal Experimentation Ethics Committee of China Pharmaceutical University (Approval number: 2019-09-011). Details on the in vivo experiments including carbon tetrachloride (CCl_4_) administration and bile duct ligation (BDL)-induced liver fibrosis model are found in the Supplementary files.

### Statistical analysis

All data analysis in this study was performed using GraphPad Prism 8.0 (GraphPad Software Inc., San Diego, CA, USA). Data were performed in triplicate for multiple independent samples and were expressed as mean ± standard error of mean (SEM). Shapiro-Wilk test was used to test normality. Unpaired two-tailed Student’s *t*-test was used to compare the two different groups and one-way ANOVA with Tukey *post hoc* analysis was used to compare multiple groups. In all figures, statistical significances are shown as ^*^p < 0.05, ^**^p < 0.01, ^***^p < 0.001, ^****^p < 0.0001. Further methodological details are provided in the Supplementary files.

## Results

### EphB2 expression is upregulated during liver disease progression

To investigate the role of EphB2 expression in liver disease progression, EphB2 expression was analyzed using publicly available RNA-seq datasets of MASLD with fibrosis, liver cirrhosis and HCC patients. We found that mRNA expression of EphB2 was significantly upregulated in human fibrotic liver (GSE193066) [30], cirrhotic liver and HCC tumors (GSE25097) [31], and was positively correlated with the progression of liver dysfunction (Fig. S1A). Next, we examined the gene expression of EphB2 and several representative profibrotic protein markers during the progression of CCl_4_-induced liver fibrosis in mice (GSE222567 and GSE135462). While the expression of profibrotic genes, such as collagen type I alpha 1 (COL1A1), matrix metalloproteinase-2 (MMP2) and α-SMA, began to show alterations in response to 1 or 2 weeks of CCl_4_ treatment (Fig. S1B, C), the mRNA level of EphB2 exhibited significant differences after 6 weeks of CCl_4_ treatment (Fig. S1C). Then, we further evaluated the transcriptional changes of these genes at various time points after culture-induced activation of primary mouse HSCs (pHSCs, GSE173920) [32]. Although statistical differences could not be observed due to the limitations of sample size, greatly enhanced expression of EphB2 was found at 24 h in culture-activated pHSCs (Fig. S1D). Theses analyses strongly suggested significant alterations in gene expression of EphB2 during the progression phase of liver fibrosis and HSC activation.

### EphB2 is sulfenylated during HSCs activation

**Fig. 1:**
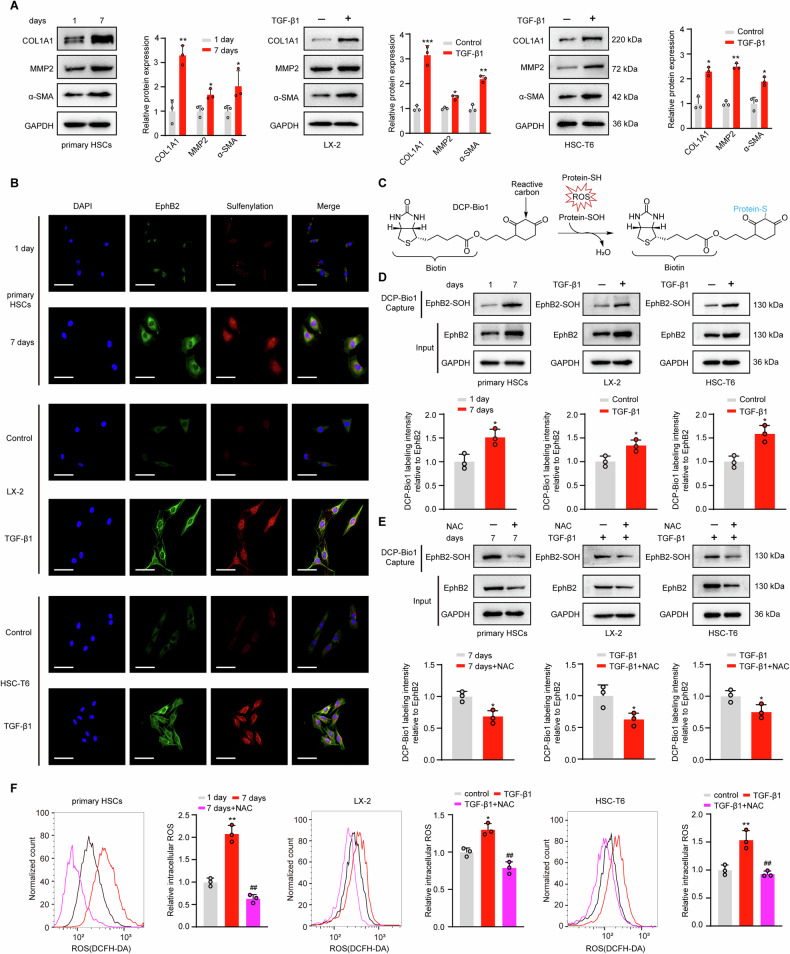
EphB2 is sulfenylated during HSCs activation. **A** Western blotting and semi-quantitative analysis of the protein expression of COL1A1, MMP2 and α-SMA during the culture-induced activation of pHSCs, and TGF-β1 (5 ng/mL)-stimulated human LX-2 and rat HSC-T6 cells (*n* = 3 per group). **B** Double immunofluorescence staining for EphB2 (green) and sulfenylation (red) in activated HSCs. Nuclei were stained with DAPI (blue) at × 400 magnification (*n* = 3 per group). Scale bar, 100 μm. **C** Schematic diagram illustrating the labeling principle of sulfenylated proteins with DCP-Bio1 probe. **D** EphB2 proteins labeled by DCP-Bio1 during the activation of HSCs (*n* = 3 per group). **E** Effects of ROS inhibitor NAC (1 mM) on the DCP-Bio1 labeling of EphB2 in HSCs (*n* = 3 per group). **F** Effects of NAC on the ROS levels in HSCs measured using DCFH-DA staining (*n* = 3 per group). × 200 magnification, scale bar, 100 μm. Data are presented as mean ± SEM. ^*^p < 0.05, ^**^p < 0.01^, ***^p < 0.001 versus control; ^##^*p* < 0.01 versus activated HSCs. The statistical analysis was analyzed using two-tailed unpaired Student´s *t*-test in **A**, **D** and **E**. Data in **F** was analyzed using one-way ANOVA with Tukey´s post hoc test.

To examine whether EphB2 could be sulfenylated during the activation of HSCs, pHSCs were isolated from C57BL/6 J mice and activated by seven days of culture, while both human LX-2 and rat HSC-T6 cells were activated by transforming growth factor β1 (TGF-β1, 5 ng/mL) stimulation. The activation of HSCs was confirmed by the enhanced expression of the profibrotic marker proteins (Fig. 1A). As expected, colocalization of EphB2 and protein sulfenylation occurred in activated HSCs (Fig. 1B). Next, a biotin-linked and dimedone based probe, DCP-Bio1 (Fig. 1C) [23], was employed to affinity capture of the sulfenic acid oxidized cellular proteins in activated HSCs. The streptavidin-agarose pull-down samples were then blotted with an EphB2-specific antibody. Immunoblotting results revealed that the amount of EphB2 proteins labeled by DCP-Bio1 (sulfenylated EphB2, designated as EphB2-SOH) were significantly increased in activated HSCs compared with the quiescent HSCs (Fig. 1D), whereas the ROS inhibitor N-acetyl-L-cysteine (NAC) reduced the sulfenylation of EphB2 (Fig. 1E) by inhibiting the production of intracellular ROS as examined by oxidation-sensitive probe DCFH-DA (Fig. 1F). These results suggest that EphB2 cysteines can be oxidized to sulfenic acid during HSCs activation.

### H_2_O_2_ mediates sulfenylation of EphB2 to enhance its kinase activity

**Fig. 2:**
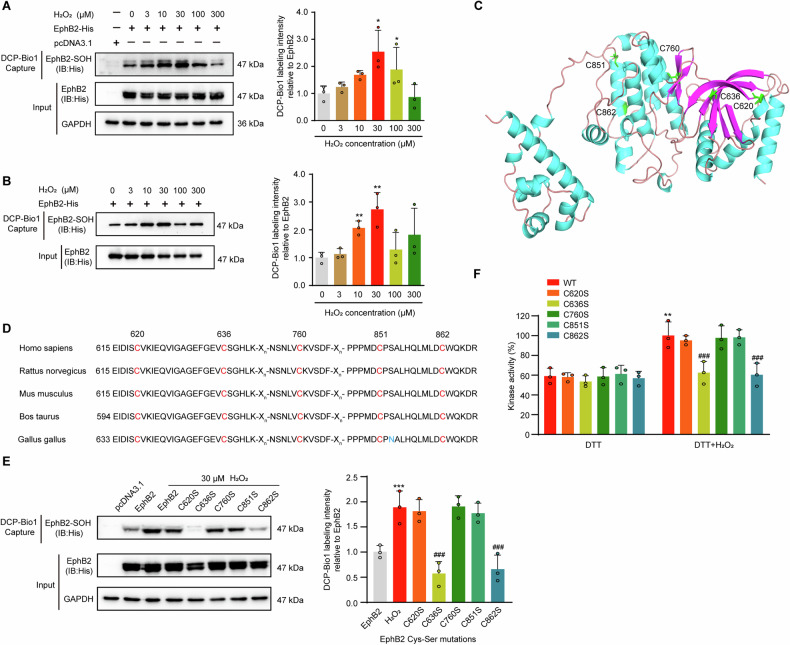
H_2_O_2_ mediates sulfenylation of EphB2 and increases its kinase activity. **A** Effects of H_2_O_2_ on the sulfenylation of EphB2. HEK293T cells were transfected with the indicated plasmids and stimulated with various concentrations of H_2_O_2_. After DCP-Bio1 labeling, sulfenylated EphB2 were identified by anti-His antibody (*n* = 3 per group). **B** Effects of H_2_O_2_ on the sulfenylation of purified recombinant EphB2 kinase domain from HEK293F cells (*n* = 3 per group). **C** Spatial localization of C620, C636, C760, C851 and C862 in the crystal structure of human EphB2 kinase domain (PDB: 3ZFM). **D** Conservation of the cysteine residues within the kinase domain of EphB2. **E** Effects of cysteine mutation on the sulfenylation of EphB2 kinase domain overexpressed in HEK293T cells (*n* = 3 per group). **F** Effects of H_2_O_2_ on the kinase activities of purified recombinant wild type and mutants of EphB2 (*n* = 3 per group). Data are presented as mean ± SEM. ^*^p < 0.05; ^**^p < 0.01^, ***^p < 0.001; ^###^*p* < 0.001. The statistical analysis was analyzed using two-tailed unpaired Student´s *t*-test in **A** and **B**. Data in **E** and **F** were analyzed using one-way ANOVA with Tukey´s post hoc test.

To further assess the effects of ROS on the cysteines sulfenylation of EphB2, HEK293T cells were transfected with His-tagged EphB2 kinase domain and treated with various concentrations of H_2_O_2_. Detection of DCP-Bio1 incorporation revealed that EphB2 proteins overexpressed in HEK293T cells could be remarkably sulfenylated by H_2_O_2_ at 30 μM (Fig. 2A). In addition, the direct induction of sulfenylation by H_2_O_2_ was also observed by using recombinant EphB2 kinase domain purified from HEK293F cells (Fig. 2B). These findings suggest that H_2_O_2_ induces sulfenylation of EphB2.

To identify the cysteine residues within EphB2 that are sulfenylated by H_2_O_2_, recombinant EphB2 kinase domain was treated with H_2_O_2_ in the presence of dimedone and subjected to liquid chromatography-tandem mass spectrometry. There are five cysteine residues (C620, C636, C760, C851, C862) in the structure of EphB2 kinase domain (Fig. 2C) and they are highly conserved across species (Fig. 2D). Among them, C862 was detected as the main site of sulfenylation modification (Fig. S2A), whereas other four cysteine sites remained unmodified (Fig. S2B). The EphB2 peptides without H_2_O_2_ treatment were also detected, and the ratio of abundance of dimedone-modified peptide ions relative to those in the unmodified version was approximately 4:1 (Fig. S2C). To test whether C862 was the only cysteine residue that is sulfenylated, individual Cys-Ser mutants of five cysteine residues in EphB2 kinase domain were constructed. Then, the changes of the mutations on EphB2 sulfenylation were evaluated after transfecting into HEK293T cells for overexpression. After the treatment with H_2_O_2_, the DCP-Bio1-labeled proteins were blotted with anti-EphB2 antibody. In addition to C862S mutant, C636S mutant also showed an unexpected decrease in sulfenylation level (Fig. 2E), suggesting that C636 might be another candidate site for functional verification. We speculated that the discrepancy between mass spectrometry and immunological assay could be due to the over-sensitivity of C636 to H_2_O_2_ oxidation, resulting in an overoxidized form with a different molecular weight. Nevertheless, these results indicated that both C636 and C862 residues of EphB2 are redox sensitive and potential sulfenylation sites.

Next, we evaluated the tyrosine kinase activity of recombinant EphB2 kinase domain using Kinase-Glo Luminescent Kinase Assay according to our previously reported method [33]. Similar to previous results of EGFR and Src, the kinase activity of wide type (WT) EphB2 was also significantly enhanced by H_2_O_2_ treatment (Fig. 2F). Although no significant kinase activity differences were observed among WT EphB2 and C620S, C760S, C851S mutants, Cys 636 and 862 mutations remarkably decreased the kinase activities of EphB2 enhanced by H_2_O_2_ (Fig. 2F), further supporting that H_2_O_2_ mediates the sulfenylation of EphB2 primarily at C636 and C862 sites to enhance its intrinsic kinase activity. To explore the possibility of changes in structural dynamics between the WT EphB2 and its mutants, molecular dynamics simulations were performed by using GROMACS software. Consistent with the similar effects of C636S and C862S mutations on the sulfenylation and kinase activities of EphB2, the trajectory of C636S mutant was closely resembled the properties observed for the C862S mutant (Fig. S3). Both mutants showed a moderate increase in the root-mean-square values which changed from 0.30 Å (WT) to 0.40 Å (C636S) and 0.39 Å (C862S), respectively. Taken together, these data suggest C636 and C862 mediate sulfenylation of EphB2 and regulate its kinase activity which may possibly through modulating the conformational dynamics of the protein.

### EphB2 sulfenylation antagonizes its ubiquitination-mediated degradation

**Fig. 3:**
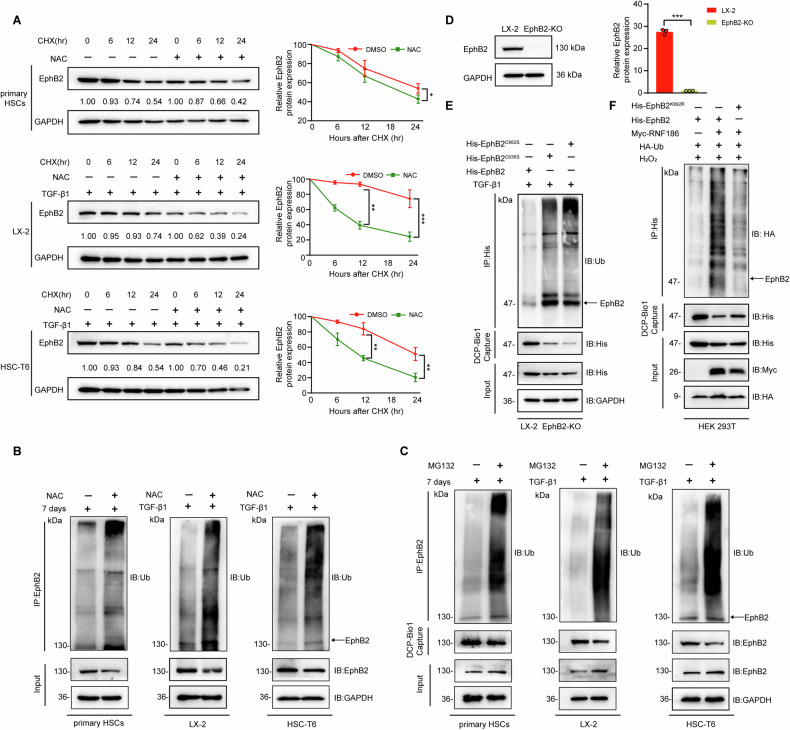
Sulfenylation of EphB2 antagonized its ubiquitination in HSCs. **A** Effects of NAC on the half-life of EphB2 protein in HSCs. Activated HSCs were treated with or without NAC (1 mM) and CHX (100 μg/mL) for the indicated periods before harvesting and western blotting (*n* = 3 per group). **B** Effects of NAC on the ubiquitination of EphB2 protein in HSCs. Activated HSCs were treated with 1 mM NAC for 24 h before harvesting and western blotting (*n* = 3 per group). **C** Effects of MG132 on the sulfenylation of EphB2 protein in activated HSCs. Activated HSCs were treated with 10 μM MG132 for 6 h before harvesting and western blotting (*n* = 3 per group). **D** Immunoblot of the full-length EphB2 in LX-2 and EphB2-KO cells (*n* = 3 per group). **E** Effects of C636S and C862S mutation of EphB2 on the ubiquitination of EphB2 kinase domain. EphB2-KO cells were activated by 5 ng/mL TGF-β1 and transfected with indicated plasmids before harvesting (*n* = 3 per group). **F** Effects of K892R mutation of EphB2 on the sulfenylation of EphB2 kinase domain in HEK293T cells. HEK293T cells were transfected with indicated plasmids, treated with H_2_O_2,_ and harvested (*n* = 3 per group). Data are presented as mean ± SEM. ^*^p < 0.05; ^**^p < 0.01^, ***^p < 0.001 versus control. The statistical analysis was analyzed using two-tailed unpaired Student´s *t*-test in **A** and **B**.

Previous studies have demonstrated that oxidation of protein cysteines can drive or compete with the subsequent ubiquitination and protein degradation [34, 35]. Our findings that both EphB2 sulfenylation and protein levels increased during liver fibrosis suggested that EphB2 sulfenylation might stabilize EphB2 protein. To examine the role of ROS in EphB2 stability, a cycloheximide (CHX, a protein synthesis inhibitor) chase assay was performed. EphB2 was revealed as a relatively stable protein during HSCs activation, with a half-life of ~24 h, whereas a rapid degradation of EphB2 at ~12 h was observed by treating HSCs with NAC (Fig. 3A), indicating that ROS stabilized EphB2 protein.

To investigate the interplay between ROS mediated sulfenylation and ubiquitination, activated HSCs were treated with NAC or proteasome inhibitor MG132. As shown in Fig. 3B, C, NAC exposure increased EphB2 poly-ubiquitination in HSCs, whereas MG132 treatment decreased sulfenylation of EphB2, indicating ROS positively regulates EphB2 protein stability by antagonizing ubiquitination-mediated proteasomal degradation. Next, to determine the role of EphB2 sulfenylation at C636 and C862 on EphB2 protein ubiquitination, we exogenously expressed WT, C636S and C862S mutants of EphB2 kinase domain in EphB2 knockout LX-2 (EphB2-KO) cells generated by CRISPR/Cas9 technique (Fig. 3D). Subsequently, the cellular ubiquitination assays demonstrated marked enhancements in the ubiquitination levels of these sulfenylation-defective mutants compared to WT EphB2 (Fig. 3E). It was reported that EphB2 can be ubiquitinated at K892 by E3 ubiquitin ligase RNF186 [36]. We then asked whether deficiency of EphB2 ubiquitination would escalate EphB2-SOH protein levels. As expected, RNF186 overexpression increased ubiquitination of WT EphB2 kinase domain but not K892R mutant (Fig. 3F). In contrast, attenuation of ubiquitination by K892R mutation significantly elevated EphB2-SOH protein levels in H_2_O_2_ stimulated HEK293T cells (Fig. 3F).

Thus, these data suggested that ROS mediated sulfenylation of EphB2 at C636 and C862 increased the stability of EphB2 through antagonizing ubiquitination-mediated degradation, which suggests a previously unrecognized way for maintaining a high level of EphB2 proteins in activated HSCs.

### Sulfenylation of EphB2 promotes HSCs activation and migration via FAK/MAPK signaling

**Fig. 4:**
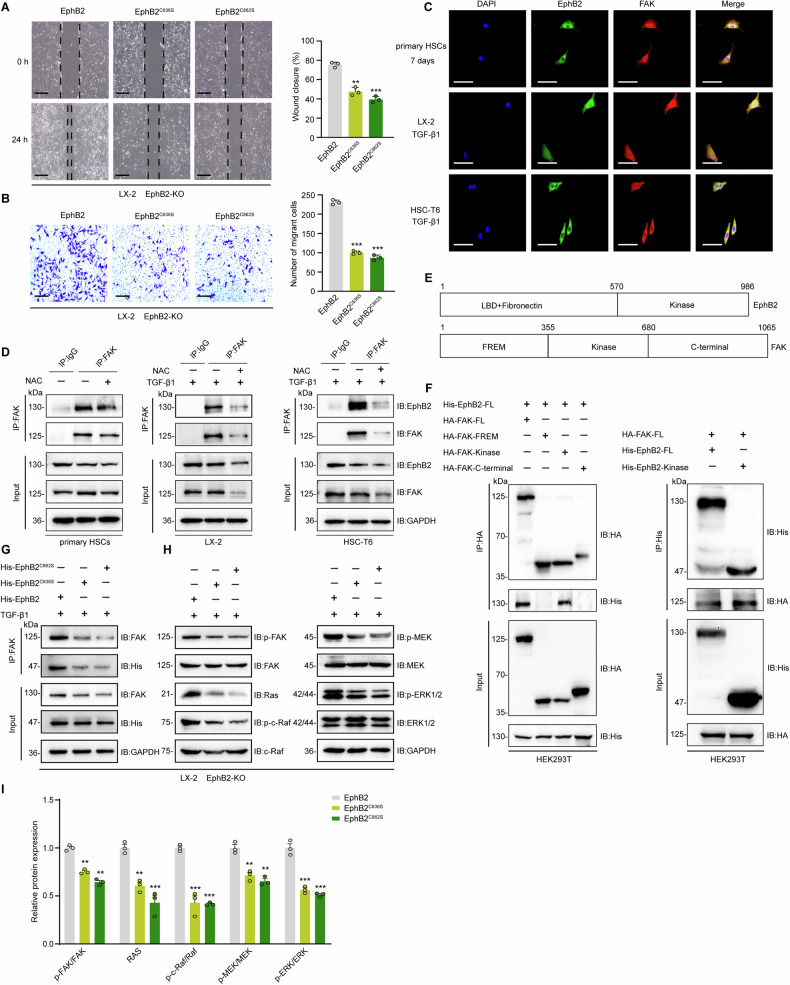
Sulfenylation of EphB2 antagonized its ubiquitination in HSCs. **A** Scratch/wound healing assay showed the migration of EphB2-KO cells transfected with WT, C636S, or C862S EphB2 variant (*n* = 3 per group), × 40 magnification, scale bar, 100 μm. **B** Transwell assay showed the migration of EphB2-KO cells transfected with WT, C636S, or C862S EphB2 variant (*n* = 3 per group), × 40 magnification, scale bar, 100 μm. **C** Immunofluorescence colocalization of EphB2 and FAK in the activated HSCs (*n* = 3 per group), × 400 magnification, scale bar, 50 μm. **D** Effects of NAC on the interactions between EphB2 and FAK. Activated HSCs were treated with or without 1 mM NAC for 24 h and harvested for immunoprecipitation (*n* = 3 per group). **E** Schematic showing full-length (FL) and truncated FAK and EphB2. **F** Co-IP analysis for the protein domains responsible for the EphB2/FAK interaction in HEK293T cells (*n* = 3 per group). **G–I** Co-IP analysis for the interaction between FAK and EphB2, western blotting assays and quantitative analysis of phosphorylated and total FAK, Ras, c-Raf, MEK, ERK1/2 in EphB2-KO cells transfected with WT, C636S, or C862S EphB2 variant at 24 h after TGF-β1-stimulation (*n* = 3 per group). Data are presented as mean ± SEM. ^**^p < 0.01, ^***^p < 0.001. The statistical analysis was analyzed using two-tailed unpaired Student´s *t*-test in **A**, **B** and **I**.

During the culture of TGF-β1-stimulated EphB2-KO cells, significant decrease in migration and invasion were observed in cells transfected with C636 or C862 EphB2 variant compared with WT EphB2 transfected cells as measured by scratch/wound healing (Fig. 4A) and transwell assays (Fig. 4B). To gain further insights into the potential effects of EphB2 sulfenylation on HSCs activation, the EphB2/focal adhesion kinase (FAK) interactions were explored due to their reported interactions and the involvement of FAK in cell migration and MASH [37, 38]. The direct interaction of EphB2 with FAK was firstly detected by immunofluorescence colocalization analysis (Fig. 4C) and further confirmed by co-immunoprecipitation (Co-IP) assay in activated HSCs (Fig. 4D). Notably, NAC treatment strongly disrupted their interactions, suggesting a positive role of sulfenylation for their interaction in HSCs (Fig. 4D). Molecular mapping with the truncated EphB2 and FAK (Fig. 4E) suggested that their intracellular kinase domains (residues 570–986 of EphB2 and residues 355–680 of FAK) were the main fragments responsible for their direct interaction (Fig. 4F). Since mitogen-activated protein kinase (MAPKs) are well-known downstream signaling molecules of Eph receptors and FAK activation, the effects of EphB2 sulfenylation on the activation of FAK/MAPK pathway were examined. As shown in Fig. 4G, C636 or C862 EphB2 variant rather than WT EphB2 retards FAK phosphorylation, thereby suppressing subsequent c-Raf/MEK/ERK phosphorylation in EphB2-KO cells (Fig. 4H, I). These results indicate that deficiency of EphB2 sulfenylation leads to a significant reduction in migration potentials of HSCs partially by disruption of EphB2/FAK interaction and inactivation of the MAPK signaling.

### Idebenone inhibits the activation of HSCs through its antioxidant activity to suppress EphB2-mediated FAK/MAPK pathway

**Fig. 5:**
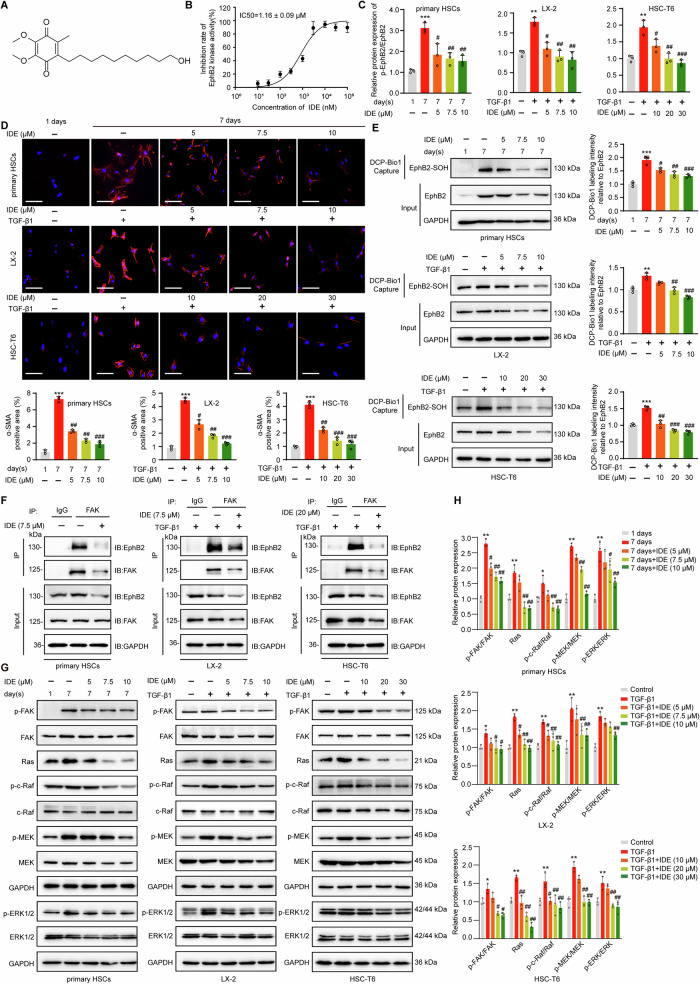
Idebenone inhibits activation of HSCs through EphB2 mediated FAK/MAPK pathway. **A** Chemical structure of IDE. **B** Inhibition curves of IDE for EphB2 protein kinase. **C** Effects of IDE on the phosphorylation of EphB2 (*n* = 3 per group). **D** Immunofluorescence staining showing the effects of IDE on the expression of α-SMA (Red) in activated HSCs. Nuclei were stained with DAPI (blue) at × 200 magnification (scale bar, 100 μm). Quantitative results of α-SMA were shown in the panel below (*n* = 3 per group). **E** Effects of IDE on the sulfenylation of EphB2 in activated HSCs (*n* = 3 per group). **F** Effects of IDE on the interactions between EphB2 and FAK (*n* = 3 per group). **G** Effects of IDE on the activation of MAPKs in HSCs (*n* = 3 per group). **H** Quantitative results of the activation of MAPKs based on the Western blotting in **G**. Data are presented as mean ± SEM. ^*^p < 0.05, ^**^p < 0.01 ^, ***^p < 0.001 versus control; ^#^*p* < 0.05, ^##^*p* < 0.01, ^###^*p* < 0.001 versus activated HSCs. Data in **C**, **D**, **E** and **H** were analyzed using one-way ANOVA with Tukey´s post hoc test.

Considering the great attraction of drug repurposing, we wished to determine if any marketed agents with antioxidant activity can inhibit the expression, sulfenylation or kinase activity of EphB2 during the activation of HSCs. Results of in silico molecular docking analysis identified that Idebenone (IDE, Fig. 5A), an analog CoQ10 [39], exhibited potential interactions with EphB2 kinase domain via hydrogen bonding (Fig. S4A). Next, in vitro EphB2 kinase assay confirmed that IDE is an EphB2 inhibitor with an IC_50_ of 1.16 ± 0.09 μM (Fig. 5B). Although IDE showed weak cellular inhibitory activities for HSCs (IC_50_ ranging from 35 to 60 μM at 24 h, Fig. S4B), it efficiently suppressed the phosphorylation of EphB2 (Fig. 5C), and the expression of α-SMA (Fig. 5D) and several other profibrogenic markers (Fig. S4C) at non-toxic doses in HSCs, demonstrating its potent inhibitory effects on HSCs activation. Subsequently, we examined the effects of IDE on the EphB2-mediated signaling in HSCs. Coincident with previous observations for EphB2 redox active cysteines mutants, IDE dose-dependently decreased the sulfenylation of EphB2 (Fig. 5E), EphB2/FAK interactions (Figs. 5F and S5), and the activation of MAPKs (Fig. 5G) in the activated HSCs. We further asked whether the antioxidant and anti-inflammatory effects of IDE participated in the inhibition of HSCs activation. We found IDE markedly downregulated the intracellular ROS and malondialdehyde levels (MDA), and concurrently upregulated the total antioxidant capacity (TAC) and activities of antioxidant enzymes such as superoxide dismutase (SOD) in a dose-dependent manner, confirming the protective roles of IDE against free radicals and lipid peroxidation during the activation of HSCs (Fig. S6A–C). Moreover, IDE significantly inhibited the proinflammatory gene expression of TNF-α, IL-6 and MCP-1 in TGF-β1-induced LX-2 cells but not in EphB2-KO cells, which indicated the direct involvement of EphB2 in the anti-inflammatory properties of IDE (Fig. S6D). These data show the great potentials of IDE in regulating HSCs activation, and the mode of action of IDE in HSCs might be linked with its antioxidant activity and the suppression of EphB2 mediated FAK/MAPK signaling.

### IDE treatment attenuates CCl_4_-induced liver fibrosis in mice

**Fig. 6:**
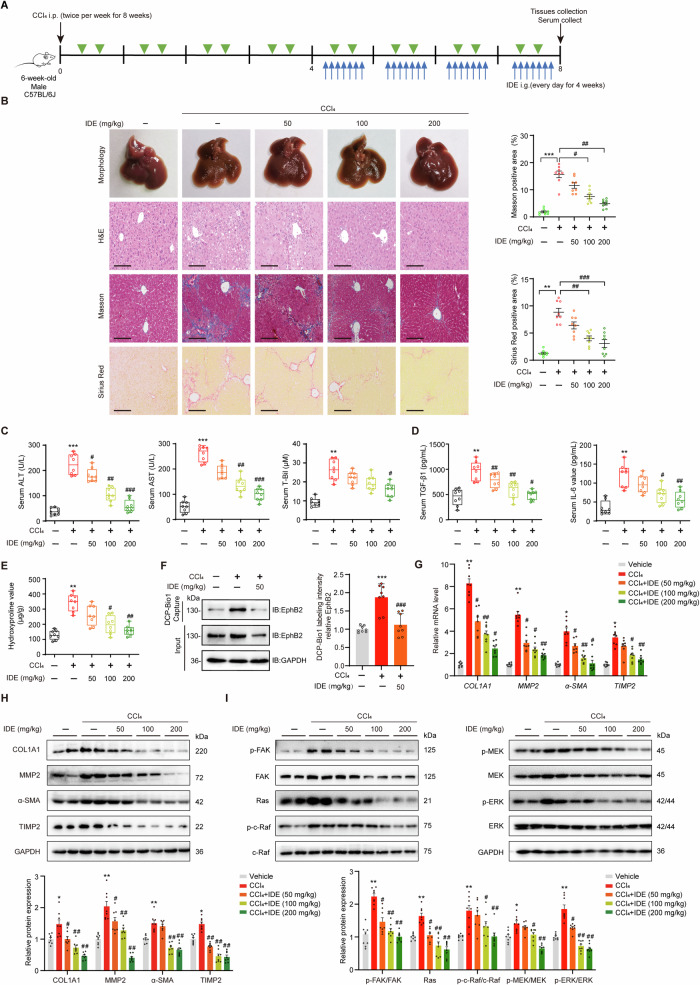
IDE treatment attenuates CCl_4_-induced liver injury and hepatic fibrosis. **A** Experimental design of the CCl_4_-induced liver fibrosis model. **B** Gross morphology of livers, H&E, Masson’s trichrome and Sirius red staining of liver samples from each group (*n* = 8 mice per group), × 200 magnification, scale bar, 100 μm. The right panels show the quantitative analyses of Masson’s trichrome and Sirius red staining. **C** Serum ALT, AST and T-Bil levels (*n* = 8 mice per group). **D** Serum TGF-β1 and IL-6 levels (*n* = 8 mice per group). **E** Hepatic hydroxyproline content (*n* = 8 mice per group). **F** Effects of IDE on the sulfenylation of EphB2 in mice liver samples (*n* = 8 mice per group). **G**, **H** Effects of IDE on the mRNA and protein expression of profibrotic markers (*n* = 8 mice per group). **I** Effects of IDE on the expression and phosphorylation of MAPKs (*n* = 8 mice per group). Data are presented as mean ± SEM. ^*^p < 0.05, ^**^p < 0.01^, ***^p < 0.001 versus vehicle group; ^#^*p* < 0.05, ^##^*p* < 0.01, ^###^*p* < 0.001 versus CCl_4_ group. Data in **B**–**I** were analyzed using one-way ANOVA with Tukey´s post hoc test.

We next analyzed the antifibrotic effects of IDE in extensively used model of liver fibrosis induced by CCl_4_ administration. IDE (0, 50, 100 or 200 mg/kg) were intragastrically administered daily for the last 4 weeks during the 8 weeks of twice-weekly intraperitoneal CCl_4_ injections (Fig. 6A). Gross morphology, H&E, Masson, Sirus red staining (Fig. 6B) revealed that IDE treatment markedly mitigated hepatic inflammatory cell infiltration and reduced collagen accumulation in CCl_4_-induced mice. In addition, IDE treatment reduced the serum levels of alanine transaminase (ALT), aspartate transaminase (AST), total-bilirubin (T-Bil), and hydroxyproline in liver tissues in a dose-dependent manner (Fig. 6C, E). Moreover, IDE reduced the expression of proinflammatory cytokines including TGF-β1 and IL-6 (Fig. 6D), possibly through the inhibition of the proinflammatory roles of EphB2 in fibrogenesis [14, 15, 18]. In line with the findings in HSCs, IDE treatment markedly suppressed the sulfenylation of EphB2 (Fig. 6F), the mRNA and protein expression of profibrogenic markers, including COL1A1, MMP2, α-SMA and TIMP2 (Fig. 6G, H), and the activation of FAK/MAPKs (Fig. 6I) induced by CCl_4_ in mouse model. It is noteworthy to mention that IDE treatment did not exert any significant cytotoxicity to other tissues, such as, heart, spleen, lung, and kidney as assessed by histological examination (Fig. S6A), and no hepatotoxicity was observed in mice treated with higher doses of IDE (300 or 400 mg/kg) once daily for 4 weeks (Fig. S7B). Taken together, these results reveal that IDE has potential to against the progression of CCl_4_-induced liver fibrosis in mice.

### IDE treatment improves BDL-induced liver fibrosis in mice

**Fig. 7:**
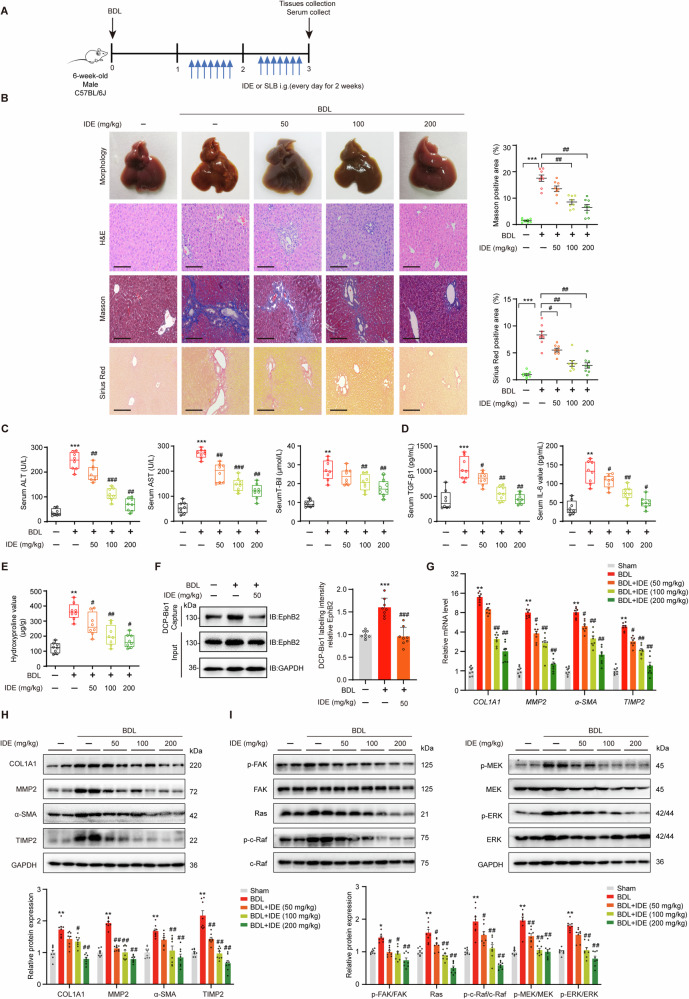
IDE markedly inhibited hepatic injury and fibrosis in BDL mice. **A** Experimental design of the BDL induced liver fibrosis. **B** Gross morphology of livers, H&E, Masson’s trichrome and Sirius red staining and quantitative analysis of liver tissue samples from various treatment groups (*n* = 8 mice per group), × 200 magnification, scale bar, 100 μm. **C** Serum ALT, AST and T-Bil levels (*n* = 8 mice per group). **D** Serum TGF-β1 and IL-6 levels (*n* = 8 mice per group). **E** Liver hydroxyproline content (*n* = 8 mice per group). **F** Effects of IDE on the sulfenylation of EphB2 in mice liver samples (*n* = 8 mice per group). **G**, **H** Effects of IDE on the mRNA and protein expression of profibrotic markers (*n* = 8 mice per group). **I** Effects of IDE on the expression and phosphorylation of MAPKs (*n* = 8 mice per group). Data are presented as mean ± SEM. ^*^p < 0.05, ^**^p < 0.01^, ***^p < 0.001 versus sham group; ^#^*p* < 0.05, ^##^*p* < 0.01, ^###^*p* < 0.001 versus BDL group. Data in **B–I** were analyzed using one-way ANOVA with Tukey´s post hoc test.

Furthermore, we established BDL-induced hepatic injury mouse model to further explore the antifibrotic effect of IDE. Different concentrations of IDE were administered daily for 2 weeks after 7 days of BDL surgery (Fig. 7A). The results showed that the collagen fiber deposition, serum transaminase and cytokines levels in BDL-treated mice were significantly increased when compared to the sham group, but were markedly decreased by IDE treatment in a dose-dependent manner, which demonstrated that IDE can confer protection against hepatic injury (Fig. 7B–E). The involvement of EphB2 sulfenylation in the anti-hepatic fibrosis activity of IDE was then investigated. As expected, IDE treatment dose-dependently decreased the amount of DCP-Bio1-labeled EphB2 (Fig. 7F), the mRNA and protein expression of profibrogenic markers (Fig. 7G, H), and the activation of FAK/MAPKs (Fig. 7I) in BDL-induced fibrotic liver tissues. Meanwhile, H&E staining showed that no histological abnormalities were detected in major organs of all IDE treated mice (Fig. S8). Furthermore, BDL caused approximately 50% mortality at 17 days after surgery, whereas IDE administration greatly improved animal survival (Fig. S9). Collectively, all these data suggested that IDE could be a potential candidate for the treatment of liver fibrosis.

## Discussion

Aberrant PTMs of proteins, such as, glycosylation, acetylation, ubiquitination, neddylation, SUMOylation and other ubiquitin-like PTMs, have been evidenced in diverse preclinical animal models of liver fibrosis [40–43]. Inhibition of these PTMs shows a promising antifibrosis efficacy when applied alone or in combination with other therapeutic approach [44, 45]. In contrast to other PTMs, the understanding of reversible cysteine oxidative PTMs in fibrotic diseases remains relatively rudimentary despite the widely recognition of the profibrotic role for ROS in liver fibrogenesis. Herein, we disclosed the hepatic sulfenylation of EphB2 during the progression of liver fibrosis in both activated HSCs and animal models, and identified the sulfenylation cysteine sites on EphB2. Our finding of EphB2 sulfenylation is supported by a recent quantitative redox proteomics investigation in which EphB2 was identified with reversible oxidation in both human cells and mouse [46]. Although the types of oxidative PTMs were not specified in that work, current studies further demonstrated sulfenylation as a new means for functional regulation of EphB2, which will provide chances for precise modulation of EphB2 activities under various pathophysiological conditions.

Cysteine oxidative PTMs mainly include reversible PTMs (such as sulfenylation, glutathionylation and nitrosylation) and irreversible PTMs (such as sulfonation, nitration and carbonylation) [47]. Crosstalk between oxidative PTMs and other PTMs can dynamically regulate the structures, activities, and functions of target proteins. For instance, sulfenylation of EGFR and Src increases their phosphorylation and kinase activities [25–28], while sulfenylation and sulfonation of protein tyrosine phosphatase PTP1B changes its conformation, leading to its inactivation and ubiquitination-mediated degradation of this protein [35]. Since non-canonical ubiquitylation can also occur on cysteine residues [48], oxidative PTMs may compete with ubiquitylation in some cases, as exemplified by insig-2 and ACAT2 protein whose sulfenylation induced protein stabilization [34, 49]. Whether EphB2 can be ubiquitylated on Cys636 or Cys862 still requires further study. In addition to the biochemical and cellular validation, determining the structures of EphB2 with or without sulfenic acids will provide more information about the regulatory mechanisms of sulfenylation in EphB2 biological function.

Although numerous small molecules or proteins are currently under development for liver fibrosis, many of them show various safety or tolerability-related issues, which greatly impedes the antifibrotic drug development by increasing the development risks and time-to-market. Drug repurposing of existing agents provides a new chance to potentially accelerate this process [50]. Extensive evidence has shown the success of repurposing for the treatment of a variety of diseases, such as COVID-19 [51], cardiovascular diseases [52], Alzheimer’s disease [53], and cancer [54]. In present study, we employed computational approach to discover IDE as a potential EphB2 inhibitor and confirmed this by biochemical and cell-based experiments. The potent antifibrotic effects of IDE observed in both animals and HSCs can be explained by a combination mechanism including the antioxidant responses and inhibition of EphB2 kinase-mediated activities. However, given the lack of a structure of human full-length EphB2, we cannot rule out the possibility of the interaction of IDE with the extracellular domain of EphB2 and their detailed direct interactions still need further investigations.

IDE is a marketed drug approved by Japanese Health Authority, which has been used for the treatment of dementia and tested in many other diseases [55]. Recently, IDE has also been reported to have preventive effects in MASH by inhibiting the adaptor protein p52Shc [56]. Given the pleiotropic protective effects of IDE, it was not surprising to possess multiple targets or modes of action by IDE. Therefore, the clarification how IDE interferes the crosstalk between p52Shc and EphB2 in the treatment of MASH requires thorough investigations. In addition to the potent cytoprotective activity, the favorable safety and tolerability of IDE have been well characterized. For example, results of a Phase I/II clinical trial of IDE in patients with primary progressive multiple sclerosis reported that a daily dose of 2250 mg IDE was well tolerated [57]. Equivalent and a higher dose for mice was examined in our study, which further confirmed the safety of IDE and represents the promising potential of repurposing IDE for treatment of liver fibrosis.

**Fig. 8:**
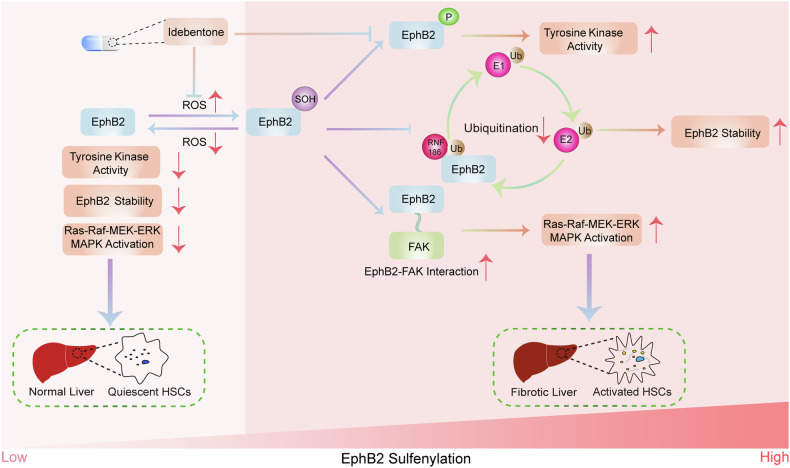
Underlying mechanisms of EphB2 regulation by sulfenylation and Idebenone during fibrosis progression.

In conclusion, we demonstrate that EphB2 undergoes sulfenylation during liver fibrosis progression, which enhances its kinase activity, antagonizes its ubiquitination-mediated degradation, promotes its interactions with other proteins, and activates the downstream FAK/MAPK signaling. We also provide evidence that IDE treatment ameliorates hepatic injury by its antioxidant activity and the inhibition of EphB2 function (Fig. 8). Thus, our findings not only advance the understanding of PTMs of EphB2 but also provide a potentially effective strategy for the treatment of liver fibrosis.

### Supplementary information

Supplementary Material and Methods

Original Western Blots

Reproducibility Checklist

## Data Availability

The data that support the findings of this study are available from the corresponding author upon reasonable request.
