# Supplementary material for: Cysteine sulfenylation contributes to liver fibrosis via the regulation of EphB2-mediated signaling
Source: Cell Death Dis. 2024 Aug 20;15(8):602. doi: 10.1038/s41419-024-06997-9 (PMC11335765; doi:10.1038/s41419-024-06997-9)
Supplement: Supplementary file 1 — Supplementary Material and Methods [file 41419_2024_6997_MOESM1_ESM.docx]

Supporting Information

**Cysteine Sulfenylation Contributes to Liver Fibrosis *via* the Regulation of EphB2-mediated Signaling**

Yueqing Han^1^, Qi Gao^1^, Yating Xu^1^, Ke Chen^1^, Rongxin Li^1^, Weiran Guo^1^ and Shuzhen Wang^1,*^

Y. Han, Q. Gao, Y. Xu, K. Chen, R. Li, W. Guo, S. Wang

School of Life Science and Technology

China Pharmaceutical University

Nanjing 211198, China

E-mail: shuzhenwang@cpu.edu.cn

**Table of contents**

[Experimental Section 3](#_Toc153022052)

[Supplementary Tables 11](#_Toc153022053)

[Supplementary Figures 13](#_Toc153022054)

[References 22](#_Toc153022055)

**EXPERIMENTAL SECTION**

**Chemicals and antibodies**

Idebenone (2,3-dimethoxy-5-methyl-6-(10-hydroxydecyl)-1,4-benzoquinone, IDE) (HY-N0303, 99.62% purity) was purchased from Med Chem Express (Shanghai, China). All the reagents were dissolved in dimethylsulfoxide (DMSO) as a stock solution. The concentration of DMSO was kept below 0.1% in the process of culturing cells to prevent any detectable effects on the cell growth. Anti-COL1A1 (#91144), anti-MMP-2 (#13132), anti-TIMP2 (#5738), anti-Ras (#8955), anti-c-Raf (#9422), anti-p-c-Raf (Ser259, #9421), anti-MEK1/2 (#9126), anti-p-MEK1/2 (Ser221, #2338), anti-p44/42 MAPK (Erk1/2, #4695), anti-p-p44/42 MAPK (p-Erk1/2, Thr202/Tyr204, #4370), anti-HA-Tag (#3724), anti-Myc-Tag (#2276), anti-His-Tag (#12698), anti-Ubiquitin (#3936) antibodies and secondary antibodies including anti-mouse IgG (#7076S) and anti-rabbit IgG (#7074S) were purchased from Cell Signaling Technology (Danvers, MA, USA). Primary antibodies against α-SMA (#ab32575), Anti-Eph receptor B1 + Eph receptor B2 (phospho Y594 + Y596, #ab61791), and species-specific secondary antibodies conjugated with Alexa Fluor-488 (#ab150105) and Alexa Fluor-594 (#ab150080) were obtained from Abcam (Cambridge, UK), anti-FAK (#A11195) and anti-p-FAK (Tyr397, #AP0302) were obtained from ABclonal (ABclonal, Wuhan, China), anti-EphB2 (#sc-130068) was obtained from Santa Cruz Biotechnology (Santa Cruz, CA, USA), and anti-GAPDH (#60004-1-Ig) was obtained from Proteintech Group (Rosemont, IL, USA), anti-Sulfenic Acid Modified Cysteine (2-Thiodimedone-Specific Ig, #ABS30) was obtained from Thermo Fisher (Thermo Scientific, USA). Recombinant human TGF-β1 was purchased from R&D Systems (Minneapolis, MN, USA).

**Cell culture, isolation of primary HSCs and treatment**

The human HSCs LX-2 and rat HSC-T6 cell lines were purchased from Procell Life Science & Technology (Wuhan, China) and Cell Bank of the Chinese Academy of Sciences (Shanghai, China), respectively, and were stimulated with 5 ng/mL TGF-β1 to induce HSCs activation. HEK293T cells were obtained from KeyGEN Biotech (Nanjing, China). HEK293F cells were a generous gift from Prof. Yibei Xiao (China Pharmaceutical University, Nanjing, China). All cell lines were authenticated by STR (Short Tandem Repeat) matching analysis and the absence of mycoplasma contamination was confirmed by using GMyc-PCR Mycoplasma Test Kit (YeaSen, Shanghai, China).

Primary HSCs was isolated from the mouse liver according to the method described previously by our group [1]. After being cultured for 7-10 days, the primary HSCs were activated and used for further research. LX-2, HSC-T6, HEK293T and primary HSCs cells were cultured in Dulbecco’s modified Eagle’s medium (DMEM) (KeyGEN Biotech, Jiangsu, China) supplemented with 10% fetal bovine serum (FBS) (TransGen Biotech, Beijing, China) and 1% penicillin-streptomycin (TransGen Biotech, Beijing, China) in a humidified 5% CO_2_ incubator at 37 ℃. HEK293F cells were maintained in OPM-293 CD05 Medium (OPM Bioscience, Shanghai, China) with 1% Penicillin-Streptomycin at 37 ℃, and 120 rpm in a humidified 5% CO_2_ shaker incubator.

**Cell viability assay**

Cell viability was evaluated using the Cell Counting Kit-8 (CCK-8, Vazyme, Nanjing, China). HSCs cells were grown to 80% confluence and then treated with IDE at the indicated concentrations for 24 h. Then, 10 μL of CCK8 reagent was added to each well and incubated for 1 h. The optical density (OD) value was measured at 450 nm using a microplate reader (Bio-Tek, USA).

**Protein stability assay**

The activated primary HSCs, LX-2 and HSC-T6 cells were incubated with 100 μg/mL cycloheximide (CHX, #A8244, Cell Signaling Technology) for the indicated time points. Cells were harvested, lysed, and assayed by Western blot using anti-EphB2 antibodies.

**Lentivirus-medicated gene knockout**

The plasmid psPAX2 (encoding HIV Gag-Pol, Addgene plasmid 12260), pMD2.G (encoding VSV-G envelope, #12259, Addgene) and LentiCRISPR-V2 (#52961, Addgene) were co-transfected in a ratio of 3:2:5, into HEK293T, by using polyethyenimine (PEI, polysciences, USA) at a 2.5:1 (w/w) PEI: DNA ratio. The supernatant medium was refreshed 24 h after transfection. After 48 and 72 h transfection, virus supernatants were harvested and filtrated with 0.22 μm filter. Target cells were infected, sorted by puromycin selection, and plated into 96-well plates at one cell per well. Isolated single clones were verified by western blot or DNA sequencing.

**Cell scratch/wound healing migration assay**

EphB2-KO LX-2 cells were seeded in a 6-well plate at a concentration of 3×10^5^ cells/well for 24 h. Cells were transfected with the indicated plasmids for 24 h when the cells reached 80% confluence, and stimulated with 5 ng/ mL TGF-β1 for 24 h. Next, an injury line was made using a 10 μL pipette tip, and the cell monolayer was washed twice with PBS. Cell migration was observed at 0 h and 24 h. The migrated areas were measured using ImageJ software.

**Transwell migration assay**

Transwell migration assay was performed using Transwell Permeable Supports (#3422, Corning, USA) in a 24-well plate. EphB2-KO LX-2 cells were transfected indicated plasmids for 24 h. The lower chambers of the plate wells were filled with DMEM supplemented with 10% FBS. Then, the cells (2×10^4^ cells/insert) in 100 μL DMEM were plated into a transwell insert and stimulated with 5 ng/mL TGF-β1, followed by adding the transwell insert into the plate well and incubating for 24 h. Subsequently, migrated cells were fixed with 4% paraformaldehyde for 20 min and were stained with 1% crystal violet dye for 5 min. Migrated cells within six separate fields of the membrane were counted under light microscope (Leica, Germany).

**Plasmid** **construction and transfection**

For *EphB2*, *FAK*, *RNF186* or *Ubiquitin* overexpression, the coding sequences (CDS) of human *EphB2*, *FAK*, *RNF186* or *Ubiquitin* were amplified and cloned into the pcDNA3.1 (+) vector (#LM1001, Lianmai, Shanghai, China). Mutagenesis was performed with the Mut Express Ⅱ Fast Mutagenesis Kit V2 (#C214, Vazyme, Nanjing, China) using primers listed in Table S1 and confirmed by sequencing. The corresponding empty vector was used as a control. Transient transfection was performed according to a standard protocol using a 2.5:1 (w/w) PEI: DNA ratio. In the co-immunoprecipitation (Co-IP) experiment, the full-length (FL) and truncated sequences of EphB2 and FAK with His or HA tag were amplified and cloned into the pcDNA3.1(+) vector. HEK293T cells were transfected with above plasmids to produce His-fused or HA-fused proteins.

**Western blotting**

Proteins were extracted from cells and liver tissues using lysis buffer (Beyotime Biotechnology, Shanghai, China) and was quantified using the BCA Assay Kit (Generay Biotech, Shanghai, China). The following procedures were performed as previously described [1].

**RNA extraction and** **quantitative real-time PCR (qRT-PCR)**

Total RNA was extracted from liver tissues using TRIzol reagent (Invitrogen, CA, USA). cDNA was synthesized from 3 μg of total RNA by Reverse Transcription Kit (Takara Japan), and qRT-PCR analysis was performed with LightCycler (Roche Diagnostics, USA) using SYBR Green Master Mix (Vazyme, Nanjing, China) according to the manufacturer’s instructions. The RNA expression level of GAPDH was used to normalize the other genes. Relative quantitation values were calculated using the 2^−△△Ct^ method. Primer sequences are listed in Table S2.

**DCP-Bio1 assay to detect sulfenylated proteins**

Sulfenylated proteins were affinity-tagged with DCP-Bio1 (Kerafast, Boston, USA) according to the previously published procedure [2]. Briefly, cells and liver tissues were homogenized in the lysis buffer which is composed of 50 mM Tris-HCl (pH 8.0), 100 mM NaCl, 100 μM DTPA, 20 mM β-glycerophosphate, 0.1% SDS, 0.5% sodium desoxycholate, 0.5% NP-40, 0.5% Triton X-100, and freshly prepared with 1mM PMSF, 10 μg/mL aprotinin, 1 mM Na_3_VO_4_, 10 mM NaF, 1 mM DCP-Bio1, 10 mM NEM, 10 mM IAM, and 200 U/mL catalase. Then, the lysates were incubated on ice for 2 h. After centrifugation at 12,000 for 10 min, the excess DCP-Bio1 was removed via a BioGel P6 spin column (Bio-Rad, USA). The protein concentration of eluants were quantified, 1 mg of protein for each sample was diluted into 2 M urea and precleared via Sepharose CL-4B beads (Sigma). Next, DCP-Bio1-bound proteins were pulled down with streptavidin-agarose beads (Thermo Scientific, USA) overnight at 4 °C with gentle rotation. Finally, the samples were assayed by Western blot using specific antibodies.

**Immunoprecipitation (IP) analysis**

Proteins were extracted from cells and quantified as described above. The samples were incubated with anti-IgG or primary antibodies in the presence of protein A agarose beads (#16-156, Millipore) overnight at 4 ℃ with gentle rotation. The agarose were washed three times with lysis buffer and precipitates were eluted with 1 × loading buffer for subsequent western blot analysis.

**Immunofluorescence analysis (IF)**

Cells were fixed with paraformaldehyde (PFA) for 30 min and permeabilized with 0.3% Triton X-100 at room temperature (RT) for 30 min. Then cells were incubated with a blocking buffer containing 5% bovine serum albumin (BSA) for 30 min, followed by incubation with 1:100 dilution of primary antibodies at 4 °C overnight. Next, the cells were washed three times with PBS, and then stained with species-specific secondary antibodies conjugated with Alexa Fluor-488 or Alexa Fluor-594 for 1 h at RT in the dark. Finally, the nucleus was stained by 4′, 6-diamidino-2-phenylindole (DAPI, sigma, USA). Final images were obtained using a fluorescence microscope (Leica, Germany). Liver tissues were fixed in 4% paraformaldehyde solution, embedded with paraffin, and cut into 4 μm-thick tissue sections which contained both the liver lobule and portal areas, and then were blocked with 5% BSA after deparaffinization, incubated with corresponding antibodies and visualized as described above.

**Liver histopathological**

Liver tissues were fixed, embedded, and cut into 4 μm-thick tissue sections as described above. Histopathological analysis was performed using hematoxylin-eosin (HE) staining, Masson trichrome Staining and Sirius Red staining Kit (Servicebio Technology, Wuhan, China) according to the standard protocols. The deposition of collagen was quantified by analyzing Masson trichrome-stained and Sirius Red stained areas using ImageJ software.

**Oxidative stress experiments**

Intracellular ROS level of cells and liver tissues was measured using oxidation-sensitive fluorescent probe 2,7-Dichlorofuorescin Diacetate (DCFH-DA) according to the manufacturer’s protocols of Reactive Oxygen Species Assay Kit (Nanjing Jiancheng Bioengineering Institute, Nanjing, China).

The total antioxidant capacity (TAC) of cells and liver tissues was detected using 2, 2′-azino-bis (3-ethylbenzthiazoline-6-sulfonic acid) (ABTS) according to the manufacturer’s protocols of Total Antioxidant Capacity Assay Kit (Beyotime, Shanghai, China).

Proteins of cells and liver tissues were extracted and quantified for detecting the Superoxide Dismutase (SOD) and Malondialdehyde (MDA) activities according to the manufacturer’s protocols of Total Superoxide Dismutase Assay Kit with NBT and Lipid Peroxidation MDA Assay Kit (Beyotime, Shanghai, China), respectively.

**Expression and purification of recombinant EphB2 proteins**

The recombinant kinase domain of EphB2 (residues 570-986) and its five Cys-to-Ser variants plasmids were transiently transfected into HEK293F cells with PEI transfection reagent as described above. After four days of transfection, cells were harvested and the recombinant proteins were purified by affinity chromatography with Ni-NTA resin (GE Healthcare, Sweden). The purity of the recombinant proteins was examined by 10% SDS-PAGE and Coomassie Brilliant Blue staining.

**Tyrosine kinase activity measurements**

Tyrosine kinase activity of recombinant kinase domain of EphB2 and Cys-to-Ser variants was analyzed using the Kinase-Glo Plus assay kit (Promega) according to the method described previously by our group [3].

**Analysis of kinase domain of EphB2 oxidation by Mass Spectrometry**

The kinase domain of EphB2 (~20 μg for dimedone experiment) buffered in 50 mM Tris-HCl, 150 mM NaCl, 0.25 mM DTT, 0.2 mM EGTA, 0.1 mM EDTA, 0.1 mM PMSF at pH 7.5 was stimulated with 30 μM H_2_O_2_ for 1 h at RT, and then reacted with 1.0 mM 5,5´-dimethyl-1,3-cyclohexanedione (dimedone, Sigma) for 10 min at 37°C in a total volume of 30 μL. The excess H_2_O_2_ was quenched with catalase.

Proteins samples were separated on 10% SDS-PAGE gels and visualized by Coomassie staining. Bands positive for kinase domain of EphB2 (~47 kDa) were excised and transferred to clean microcentrifuge tubes. Gels pieces were firstly destained twice with 200 μL of 50 mM NH_4_HCO_3_ and 50% acetonitrile and then dried twice with 200 μL of acetonitrile. Afterwards, the dried pieces of gels were incubated in ice-cold digestion solution (trypsin 12.5 ng/μL and 20 mM NH_4_HCO_3_) for 20 min and then transferred into a 37°C incubator for digestion overnight. Finally, peptides in the supernatant were collected after extraction twice with 200 μL extract solution (5% formic acid in 50% acetonitrile) and dried under the protection of N_2_.

The tryptic peptides were dissolved in 0.1% formic acid (solvent A), directly loaded onto a reversed-phase analytical column (Acclaim PepMap C18, 75 μm × 25 cm). The gradient was comprised of an increase from 2% to 30% solvent B (0.1% formic acid in 98% acetonitrile) over 50 min, 30% to 50% in 5 min and climbing to 80% in 1min then holding at 80% for the last 4 min, all at a constant flow rate of 300 nl/min on an EASY-nLC 1200 UPLC system.

The peptides were subjected to NSI source followed by tandem mass spectrometry in Orbitrap Exploris 480 MS coupled online to the UPLC. For DDA experiments, full MS resolutions were set to 60,000 at *m/z* 200 and the full MS AGC target was 300% with an IT of 50 ms (range from 350-1600).

Tandem mass spectra were extracted by Proteome Discoverer software (Thermo Fisher Scientific, version 2.4) and searched against a self-built database assuming the digestion enzyme trypsin. Oxidation of methionine and protein N-terminus acetylation of protein was specified as a variable modification, and the number of max missed cleavage sites was set to 2 with 10 ppm mass error for precursor ions. The acceptance criteria for identifications were the false discovery rate (FDR) should be less than 1% for peptides and proteins.

**CCl_4_-induced liver fibrosis**

CCl_4_-induced liver fibrosis model was established according to our previous report [4]. Mice were injected intraperitoneally with 0.1 mL of corn oil or CCl_4_ (10% solution in corn oil) /10 g body weight twice a week for 4 weeks. Then, the mice were randomly divided into six groups (n = 8): corn oil (Vehicle), CCl_4_-corn oil (CCl_4_), CCl_4_ + 50 mg/kg IDE, CCl_4_ + 100 mg/kg IDE and CCl_4_ + 200 mg/kg IDE. The mice were treated with 4 more weeks of CCl_4_ (total, 8 weeks), whereas control mice were dealt with the same volume of corn oil. After the first 4 weeks of CCl_4_ injection, IDE was resuspended in corn oil and orally administered daily for four-week treatment. The serum and liver tissues were collected after an overnight fast as described above.

**BDL-induced liver fibrosis model**

BDL was performed as previously described [5]. Briefly, mice were anesthetized and the peritoneal cavity was opened through a 2-cm midline incision. The common bile duct below the bifurcation was separated and ligated using surgical sutures, while sham-treated mice were subjected to the same procedure, exposing the choledochal duct, but without ligation. Twenty-four hours after surgery, mice receiving BDL were randomly divided into six groups (n = 10) for efficacy and survival experiments: sham operation group, BDL group, BDL + 50 mg/kg IDE, BDL + 100 mg/kg IDE and BDL + 200 mg/kg IDE. In efficacy experiments the mice were orally administered IDE each day staring on day seven after BDL operation, and the sham operation mice were dealt with the same volume corn oil for fourteen days. The serum and liver tissues were collected after an overnight fast as described above. In survival experiments, the mice were orally administered IDE daily until death.

**Serum biochemical analysis, the total cholesterol, triglyceride and hydroxyproline assay**

To analyze liver function, the serum was separated from mouse blood samples, and alanine transaminase (ALT), aspartate transaminase (AST), total bilirubin (T-Bil) in the serum and hydroxyproline in liver tissues were detected following the manufacturer’s protocols (Nanjing Jiancheng Bioengineering Institute, China). Serum levels of TGF-β1 and IL-6 were measured using ELISA kits (Dakewe Biotech, Shenzhen, China).

**Molecular docking**

Molecular docking was performed using Autodock Vina software with default parameters. First, the 3-D structure of the IDE molecule was built using the Chem3D tool of Chemoffice software of the 2019 version, then docked with the crystallographic structure of the kinase domain of EphB2 (PDB: 3ZFM), and the best five poses of the molecules were retained and scored. Both the docking results and binding pockets were visualized using PyMOL as previously reported [6].

**Molecular dynamics simulations**

Molecular dynamics simulations were performed with the GROMACS 4.5.4 program package in the Ubuntu 14.04 operating system. GROMOS 54A7 forcefield was used for energy minimization, dismissing when the maximum force was found lesser than 10 kJ/mol. Then the systems were equilibrated using two consecutive 100 ps position-restrained dynamics runs in the NVT and NPT ensembles, respectively.

Subsequently, the equilibrated systems were subjected to 8 ns production molecular dynamics runs with a time step of 10 ps. Run trajectories were obtained and with GROMACS utilities analysis was carried out. Using g_rms quality assurance of all the molecules was performed [7].

**Supplementary Tables**

**Supplementary Table S1. Primer sequences used for cloning and site-directed mutagenesis of EphB2 kinase domain**

|  | **Forward sequence (5'-3')** | **Reverse sequence (5'-3')** |
| --- | --- | --- |
| EphB2-WT | CGGGATCCGCCACCATGTTTGAGCGTGCTGACTCGGAG | CGGAATTCTTAGTGGTGATGGTGATGATGTCCCCCGCAAGAACAGTCATTGCTTTCCTT |
| EphB2-C620S | ATCTCCAGTGTCAAAATTGAGCAGGTGATCGG | TTGACACTGGAGATGTCAATTTCCTTGGCAAACTCC |
| EphB2-C636S | GTTTGGCGAGGTCAGCAGTGGCCACCTGAAGCTGCCAG | CACTGCTGACCTCGCCAAACTCCCCTGCTCCGATCACCTG |
| EphB2-C760S | CTGGTCAGCAAGGTGTCGGACTTTGGGC | ACACCTTGCTGACCAGGTTGCTGTTGACGAG |
| EphB2-C851S | ATGGACAGCCCGAGCGCCCTGCACCAACTC | GCTCGGGCTGTCCATGGGCGGTGGCAGCCG |
| EphB2-C862S | GCTGGACAGTTGGCAGAAGGACCGCAACCACCG | CTTCTGCCAACTGTCCAGCATGAGTTGGTGCAG |

**Supplementary Table 2.** **Primer sequences used for RT-qPCR**

| **Gene** | **Forward sequence (5'-3')** | **Reverse sequence (5'-3')** |
| --- | --- | --- |
| Mouse *COL1A1* | GACAGGCGAACAAGGTGACAGAG | CAGGAGAACCAGGAGAACCAGGAG |
| Mouse *EphB2* | GCGGCTACGACGAGAACAT | GGCTAAGTCAAAATCAGCCTCA |
| Mouse *MMP2* | ACCATGCGGAAGCCAAGATGTG | AGGGTCCAGGTCAGGTGTGTAAC |
| Mouse *α-SMA* | CGTGGCTATTCCTTCGTGACTACTG | CGTCAGGCAGTTCGTAGCTCTTC |
| Mouse *TIMP2* | CGCTTAGCATCACCCAGAAGAAGAG | AGTCCATCCAGAGGCACTCATCC |
| Mouse *β-actin* | GGCTGTATTCCCCTCCATCG | CCAGTTGGTAACAATGCCATGT |
| Human *TNF-α* | CCTCTCTCTAATCAGCCCTCTG | GAGGACCTGGGAGTAGATGAG |
| Human *IL-6* | ACTCACCTCTTCAGAACGAATTG | CCATCTTTGGAAGGTTCAGGTTG |
| Human *MCP-1* | CAGCCAGATGCAATCAATGCC | TGGAATCCTGAACCCACTTCT |
| Human *β-actin* | CATGTACGTTGCTATCCAGGC | CTCCTTAATGTCACGCACGAT |

**Supplementary Figures**

**
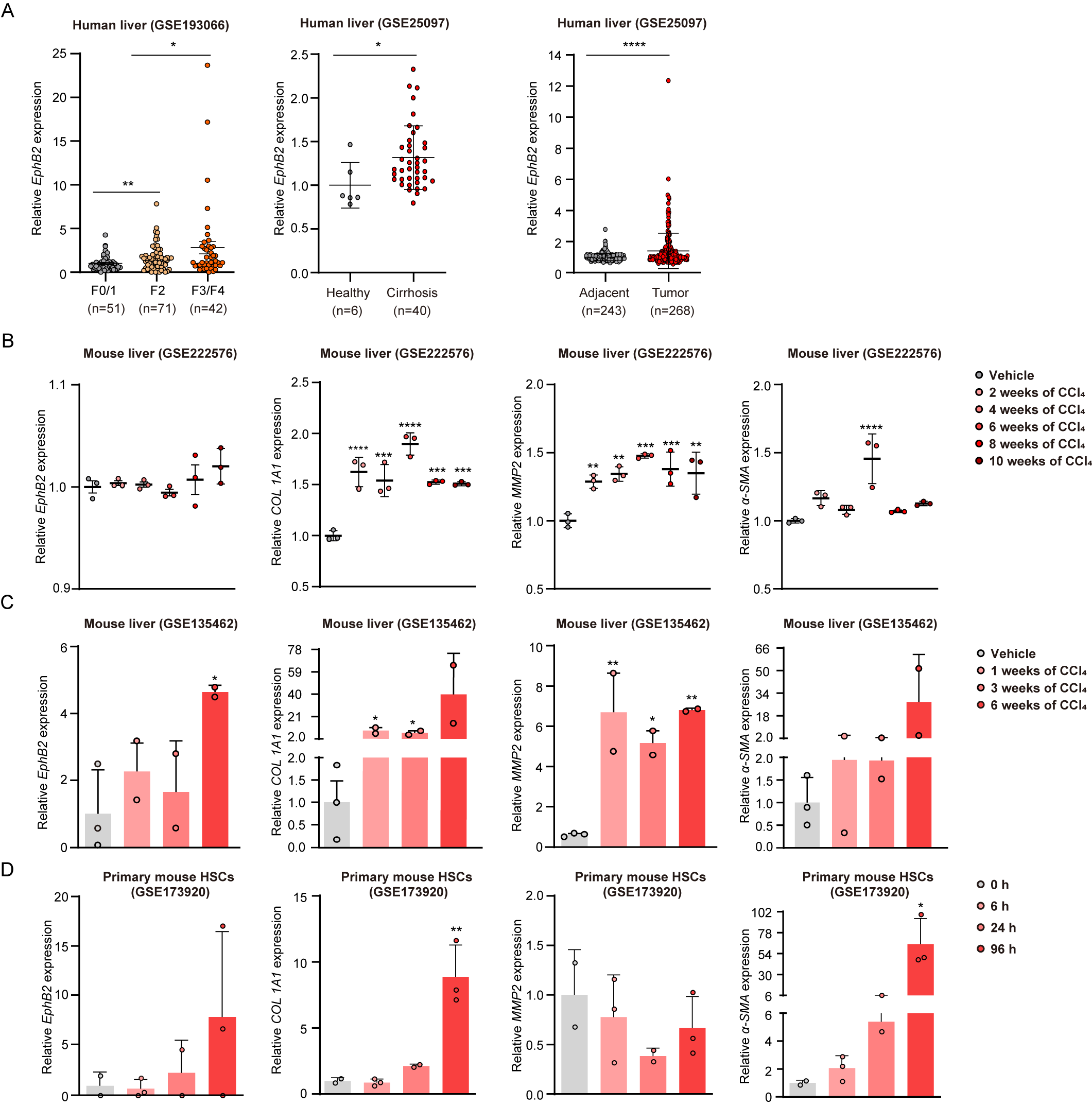
**

**Fig. S1 Expression of EphB2 during the progression of liver dysfunction. A** *EphB2* expression in patients with NAFLD at different fibrosis stages (GSE193066), Cirrhosis (GSE25097) and HCC (GSE25097). **B-C** *EphB2* and liver fibrosis-related marker genes expression during the progression of liver fibrosis induced by CCl_4_ treatment (GSE222567 and GSE135462). **D** *EphB2* and liver fibrosis-related marker genes expression at various time points after culture-induced activation of primary mouse HSCs (GSE173920)). Data are presented as mean ± SEM. ^*^*p* < 0.05, ^**^*p* < 0.01, ^***^*p* < 0.001, ^****^*p* < 0.0001 versus control.


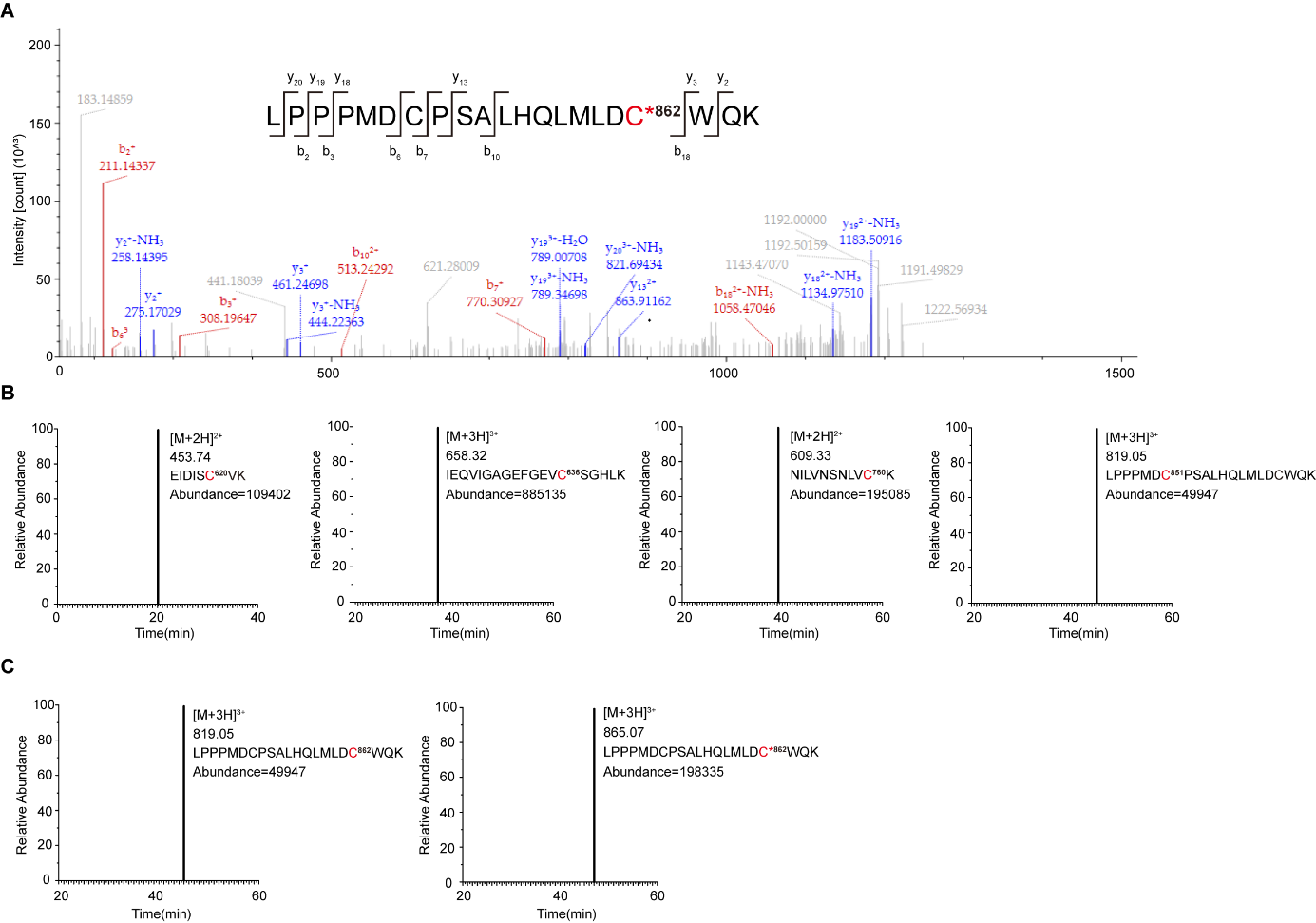


**Fig. S2** **Identification of the** **sulfinylated cysteines in EphB2 kinase domain by dimedone labeling and mass spectrometry. A** Identification of dimedone-labeled C862 of EphB2 kinase domain treated with 30 μM H_2_O_2_ for 1 h at RT. **B** The mass spectrometry data of the C620, C636, C760, C851 residues. **C** Left spectrum: Extracted ion (ion current at *m/z* 819.05 [M+3H]^3+^) chromatogram corresponding to the unmodified peptide (LPPPMDCPSALHQLMLDC^862^WQK) from EphB2 kinase domain. Right spectrum: Extracted ion (ion current at *m/z* 865.07 [M+3H]^3+^) chromatogram corresponding to the dimedone-tagged peptide (LPPPMDCPSALHQLMLDC^*862^WQK) from EphB2 kinase domain, which identifies C862 of EphB2 as a S-sulfinylated site. The ratio of dimedone-modified to unmodified peptide was approximately 4:1.


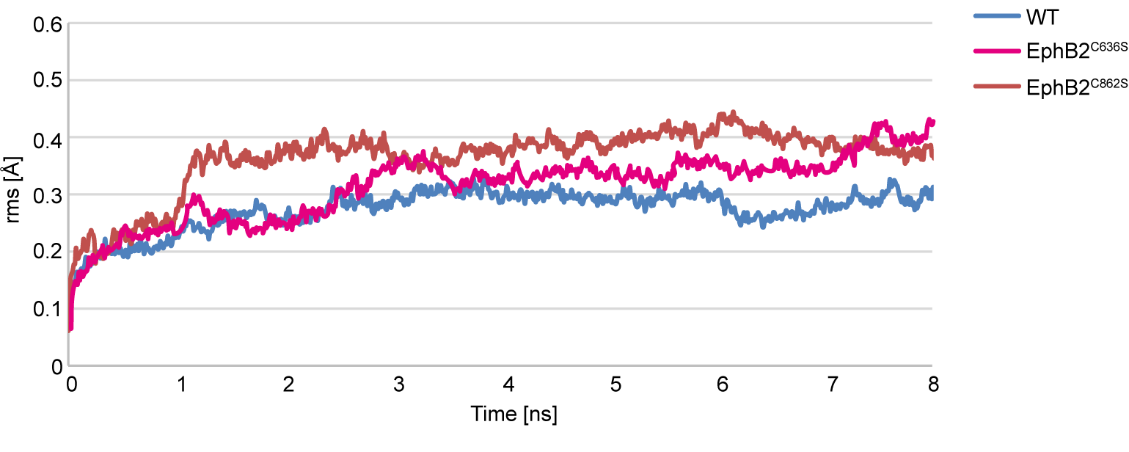


**Fig. S3 Graph comparing the root-mean-square (rms) deviation of wild-type EphB2 kinase domain (WT) and its mutants including** **C636S and C862S during the molecular dynamics simulations.**


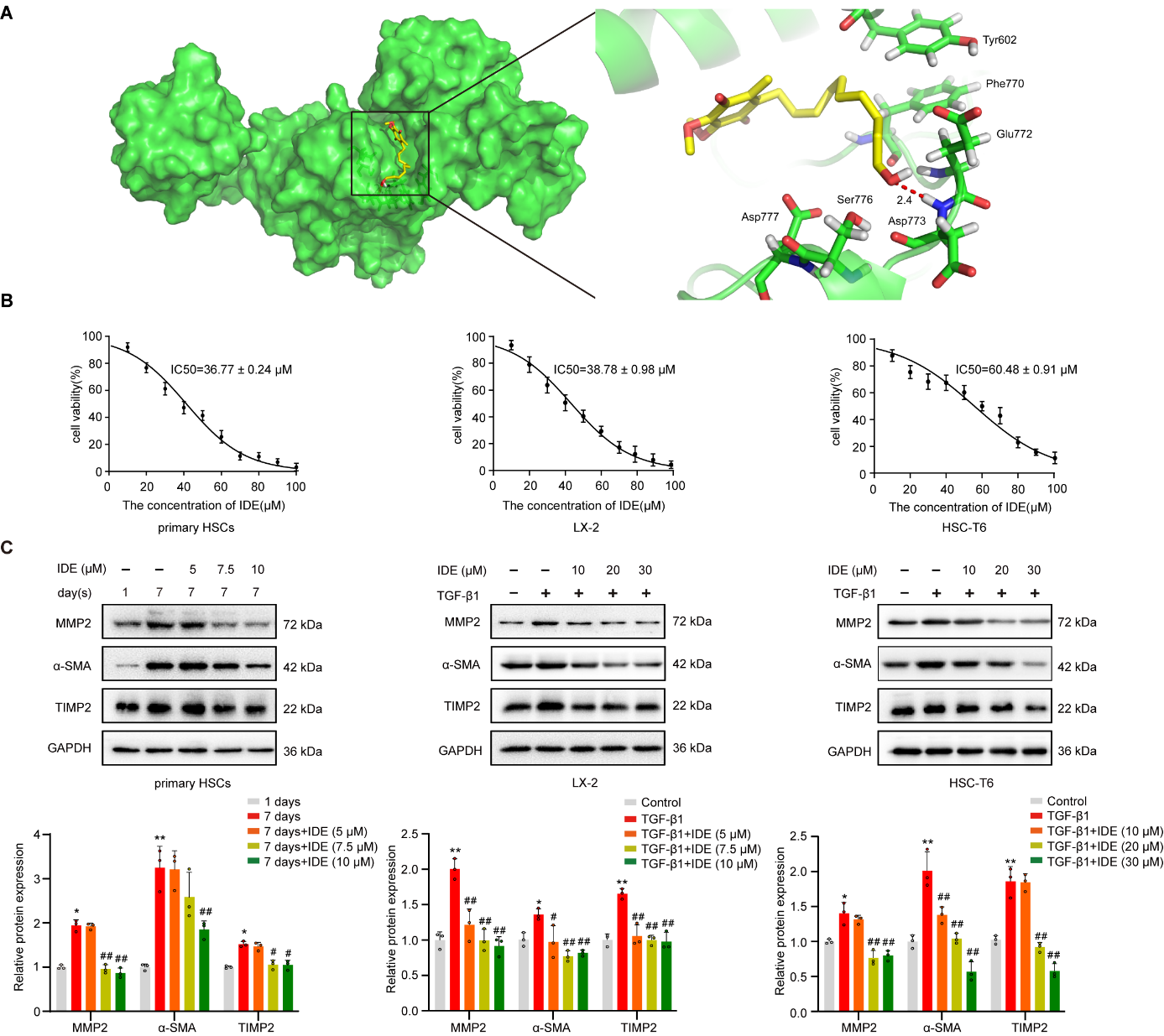


**Fig. S4 Idebenone inhibits the activation of HSCs through its antioxidant activity. A** Molecular docking analysis of the direct interaction between IDE and the kinase domain of EphB2. IDE demonstrated suitable steric complementary with the kinase domain of EphB2 with a docking score (binding energy) of -6.1 kcal/mol and one potential hydrogen bond with residue Asp773 of EphB2. **B** IC_50_ curves of IDE were determined by the CCK-8 assay in mouse primary HSCs, LX-2 cells, and HSC-T6 cells (n = 3 per group). **C** Western blot assay and quantitative analysis of protein levels of MMP2, α-SMA and TIMP2 in primary HSCs, LX-2 cells and HSC-T6 cells after treatment with IDE at concentrations indicated (n = 3 per group). Data are presented as mean ± SEM. ^*^*p* < 0.05, ^**^*p* < 0.01 versus control; ^#^*p* < 0.05, ^##^*p* < 0.01 versus activated HSCs. Data in (C) was analyzed using one-way ANOVA with Tukey´s post hoc test.


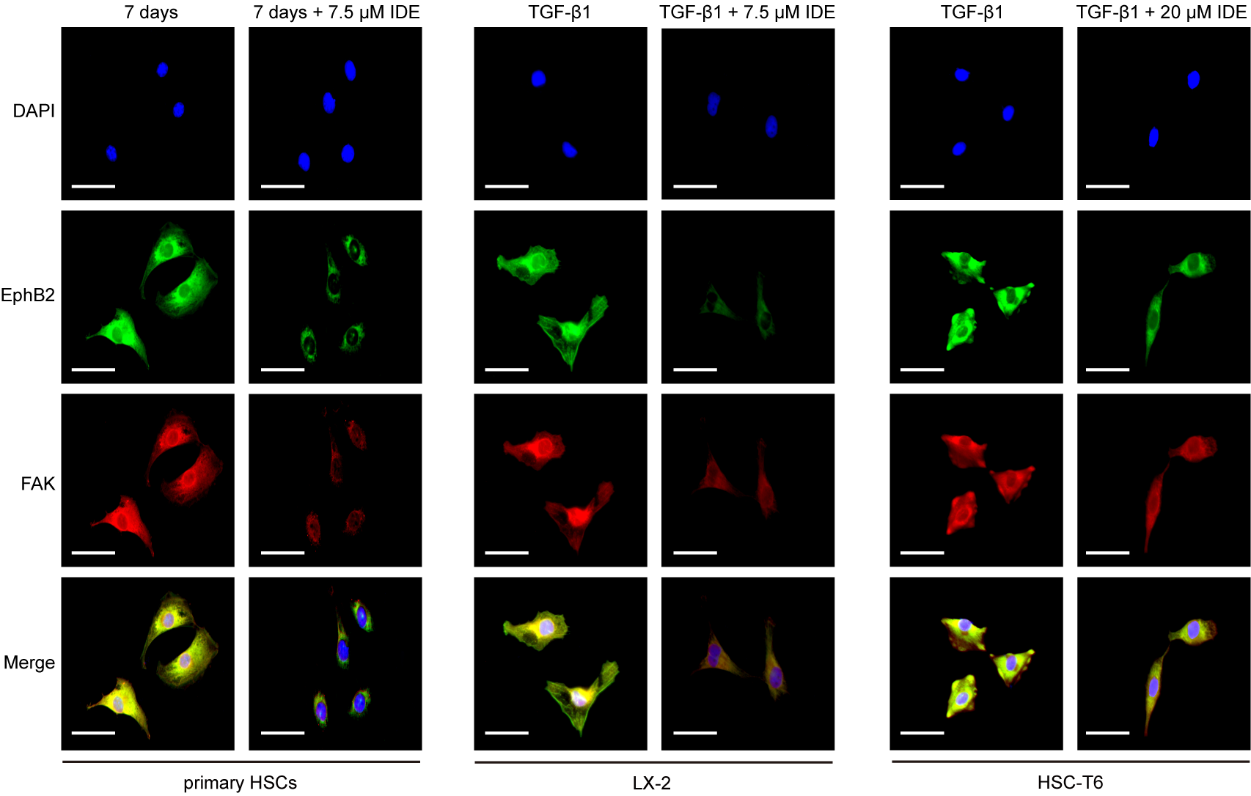


**Fig. S5 Idebenone disrupts EphB2/FAK interaction in HSCs.** The effects of IDE on the immunofluorescence colocalization of EphB2 and FAK in primary HSCs, LX-2 cells and HSC-T6 cells at 24 h after incubation with IDE at concentrations indicated (n = 3 per group) ×400 magnification, scale bar, 100 μm.


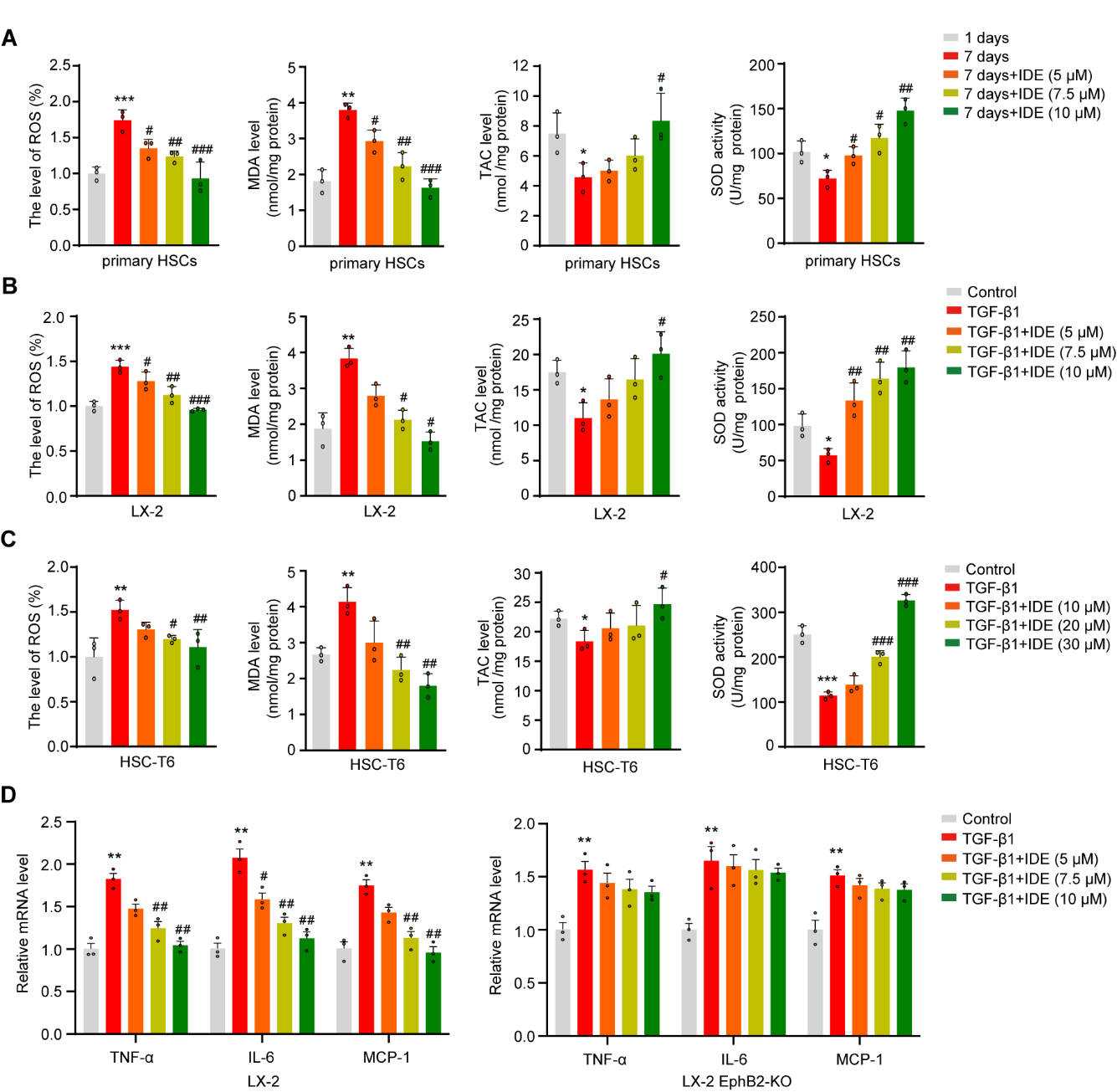


**Fig. S6 Idebenone exerts antioxidant and anti-inflammatory effects on HSCs. A-C** Effects of IDE on the levels of ROS, MDA, TAC, and SOD were assessed in primary HSCs (**A**), LX-2 cells (**B**) and HSC-T6 cells (**C**) (n = 3 per group). **D** Effects of IDE on the pro-inflammatory gene expression in LX-2 and EphB2-KO cells (n = 3 per group). Data are presented as mean ± SEM. ^*^*p* < 0.05, ^**^*p* < 0.01, ^***^*p* < 0.001 versus control; ^#^*p* < 0.05, ^##^*p* < 0.01, ^###^*p* < 0.001 versus activated HSCs. Data in (A-D) were analyzed using one-way ANOVA with Tukey´s post hoc test.


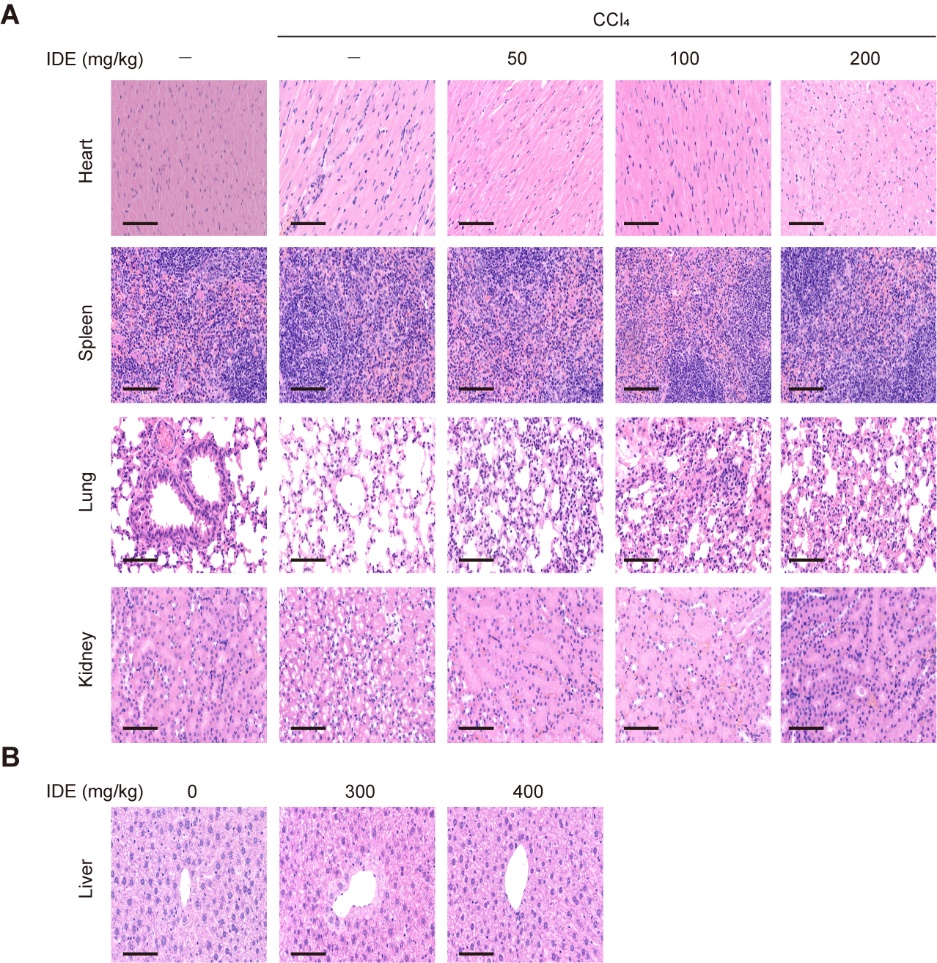


**Fig. S7 Histological examinations of the major organs from mice treated with IDE. A** H&E staining of heart, spleen, lung, and kidney tissue from CCl_4_-induced liver fibrosis mice treated with different concentrations of IDE (n = 8 mice per group). **B** C57BL/6J mice were intragastrically administered with different concentrations of IDE (0, 300 or 400 mg/kg) for four weeks (n = 8 mice per group). Histopathological images of H&E-stained liver sections were observed, ×200 magnification, scale bar, 100 μm.


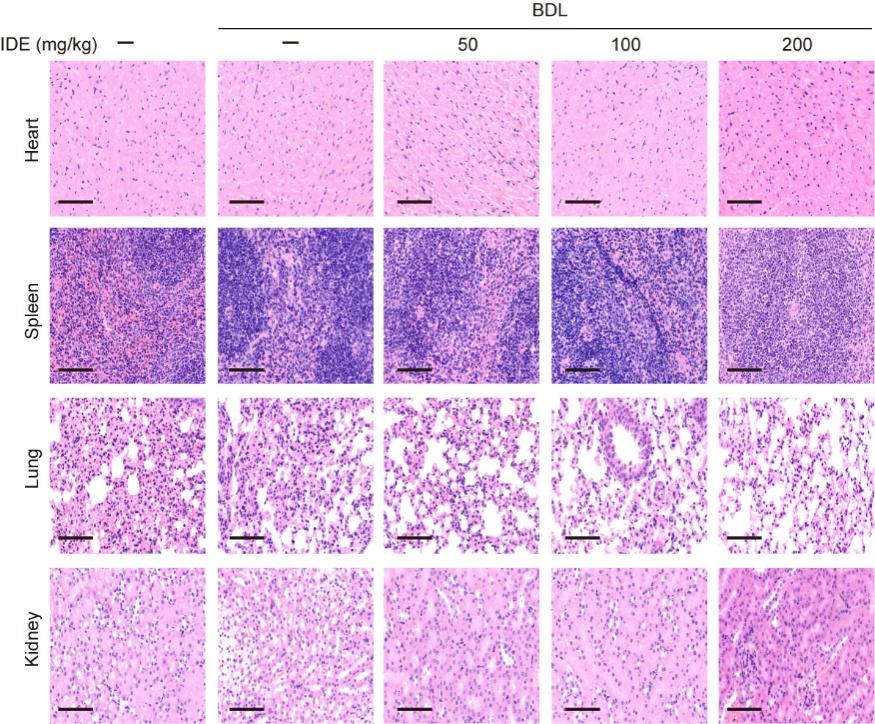


**Fig. S8 Histological examinations of the major organs from BDL-induced fibrotic mice treated with IDE.** H&E staining of heart, spleen, lung, and kidney tissue from BDL-induced fibrotic mice treated with different concentrations of IDE (n = 8 mice per group), ×200 magnification, scale bar, 100 μm.


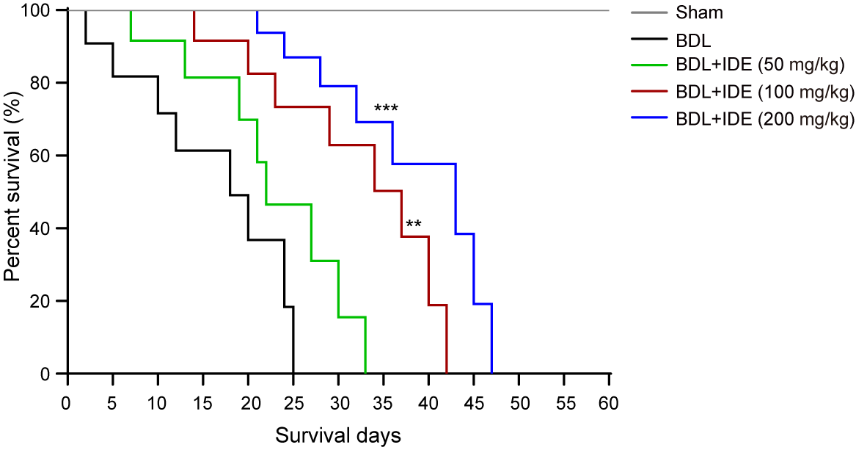


**Fig. S9: Effects of IDE on the survival in BDL mouse. Kaplan-Meier survival curve of BDL mice treated with different concentrations of IDE.** Differences between survival curves for 47 days in groups were compared using a long-rank test (n = 10 mice per group), Data are presented as mean ± SEM. ^**^ *p* < 0.01, ^***^ *p* < 0.001 versus BDL group. Data was analyzed using one-way ANOVA with Tukey´s post hoc test.

**References**

1. Chen X, Zhang D, Wang Y, Chen K, Zhao L, Xu Y, et al. Synergistic antifibrotic effects of miR-451 with miR-185 partly by co-targeting EphB2 on hepatic stellate cells. Cell Death Dis. 2020;11(5):402.

2. Klomsiri C, Nelson KJ, Bechtold E, Soito L, Johnson LC, Lowther WT, et al. Use of dimedone-based chemical probes for sulfenic acid detection evaluation of conditions affecting probe incorporation into redox-sensitive proteins. Methods Enzymol. 2010;473:77-94.

3. Wang S, Xie W, Wang D, Peng Z, Zheng Y, Liu N, et al. Discovery of a small molecule targeting SET-PP2A interaction to overcome BCR-ABLT315I mutation of chronic myeloid leukemia. Oncotarget. 2015;6(14):12128-12140.

4. Chen K, Guo W, Li R, Han Y, Gao Q, Wang S. Demethylzeylasteral attenuates hepatic stellate cell activation and liver fibrosis by inhibiting AGAP2 mediated signaling. Phytomedicine. 2022;105:154349.

5. Zhu X, Ye S, Yu D, Zhang Y, Li J, Zhang M, et al. Physalin B attenuates liver fibrosis via suppressing LAP2α-HDAC1-mediated deacetylation of the transcription factor GLI1 and hepatic stellate cell activation. Br J Pharmacol. 2021;178(17):3428-3447.

6. Seeliger D, de Groot BL. Ligand docking and binding site analysis with PyMOL and Autodock/Vina. J Comput Aided Mol Des. 2010;24(5):417-422.

7. Meier K, Schmid N, van Gunsteren WF. Interfacing the GROMOS (bio)molecular simulation software to quantum-chemical program packages. J Comput Chem. 2012;33(26):2108-2117.
