# Supplementary material for: Cysteine sulfenylation contributes to liver fibrosis via the regulation of EphB2-mediated signaling
Source: Cell Death Dis. 2024 Aug 20;15(8):602. doi: 10.1038/s41419-024-06997-9 (PMC11335765; doi:10.1038/s41419-024-06997-9)

Figure 1A

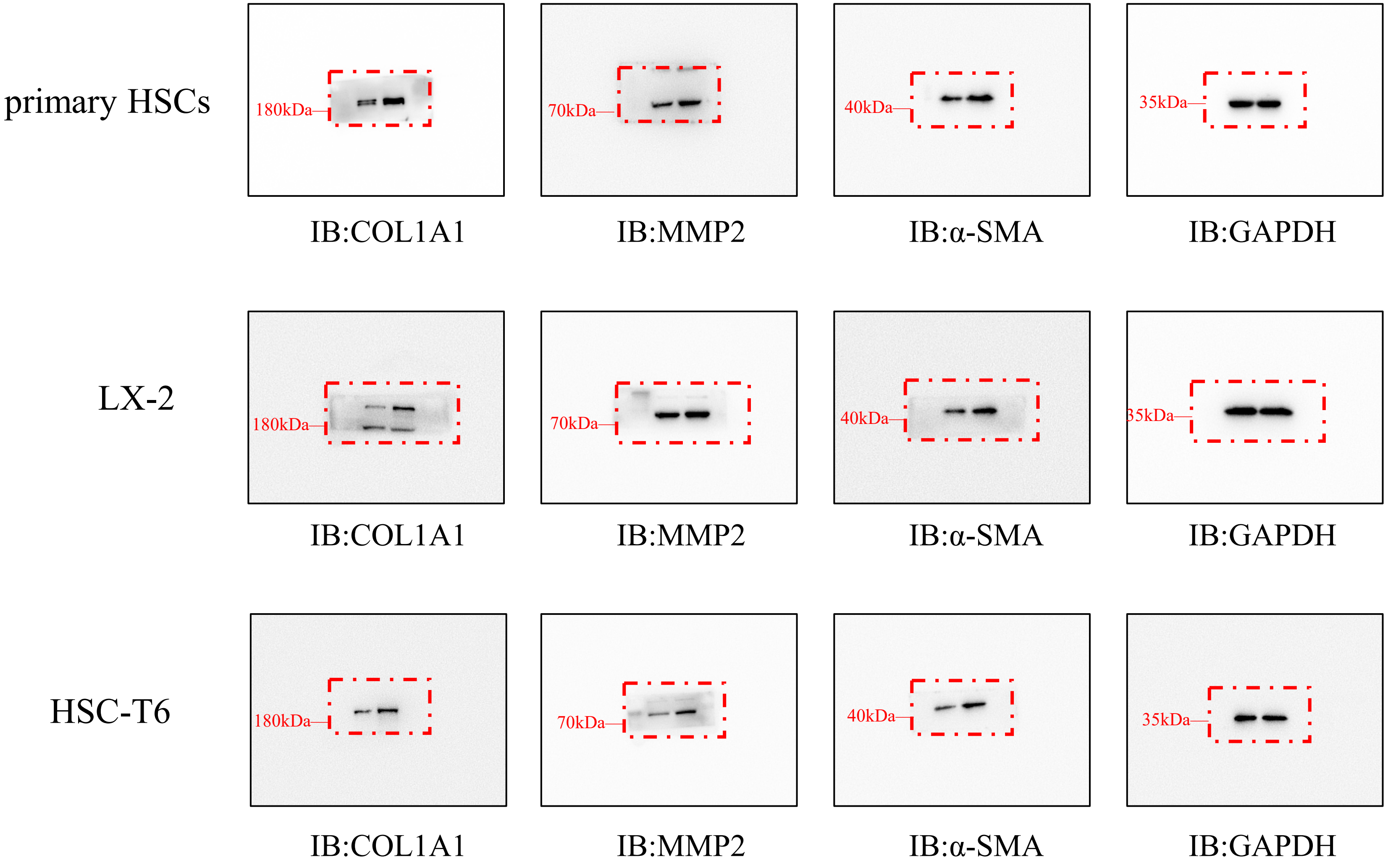
primary HSCs
LX-2
HSC-T6
180kDa
70kDa
40kDa
35kDa
IB:COL1A1
IB:MMP2
IB:α-SMA
IB:GAPDH

Figure 1D

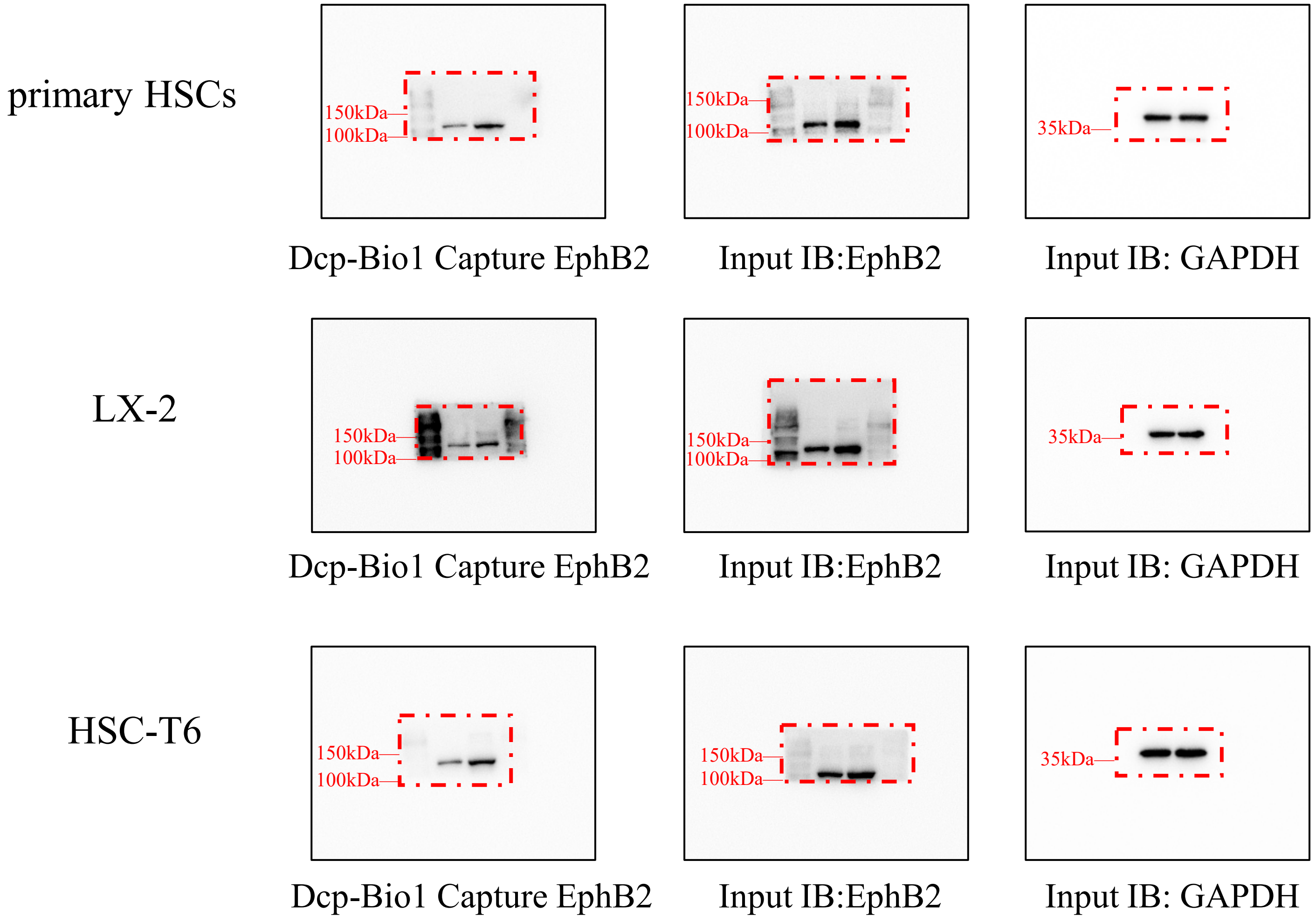
primary HSCs
150kDa
100kDa
Dcp-Bio1 Capture EphB2
150kDa
100kDa
Input IB:EphB2
35kDa
Input IB: GAPDH
LX-2
150kDa
100kDa
Dcp-Bio1 Capture EphB2
150kDa
100kDa
Input IB:EphB2
35kDa
Input IB: GAPDH
HSC-T6
150kDa
100kDa
Dcp-Bio1 Capture EphB2
150kDa
100kDa
Input IB:EphB2
35kDa
Input IB: GAPDH

Figure 1E

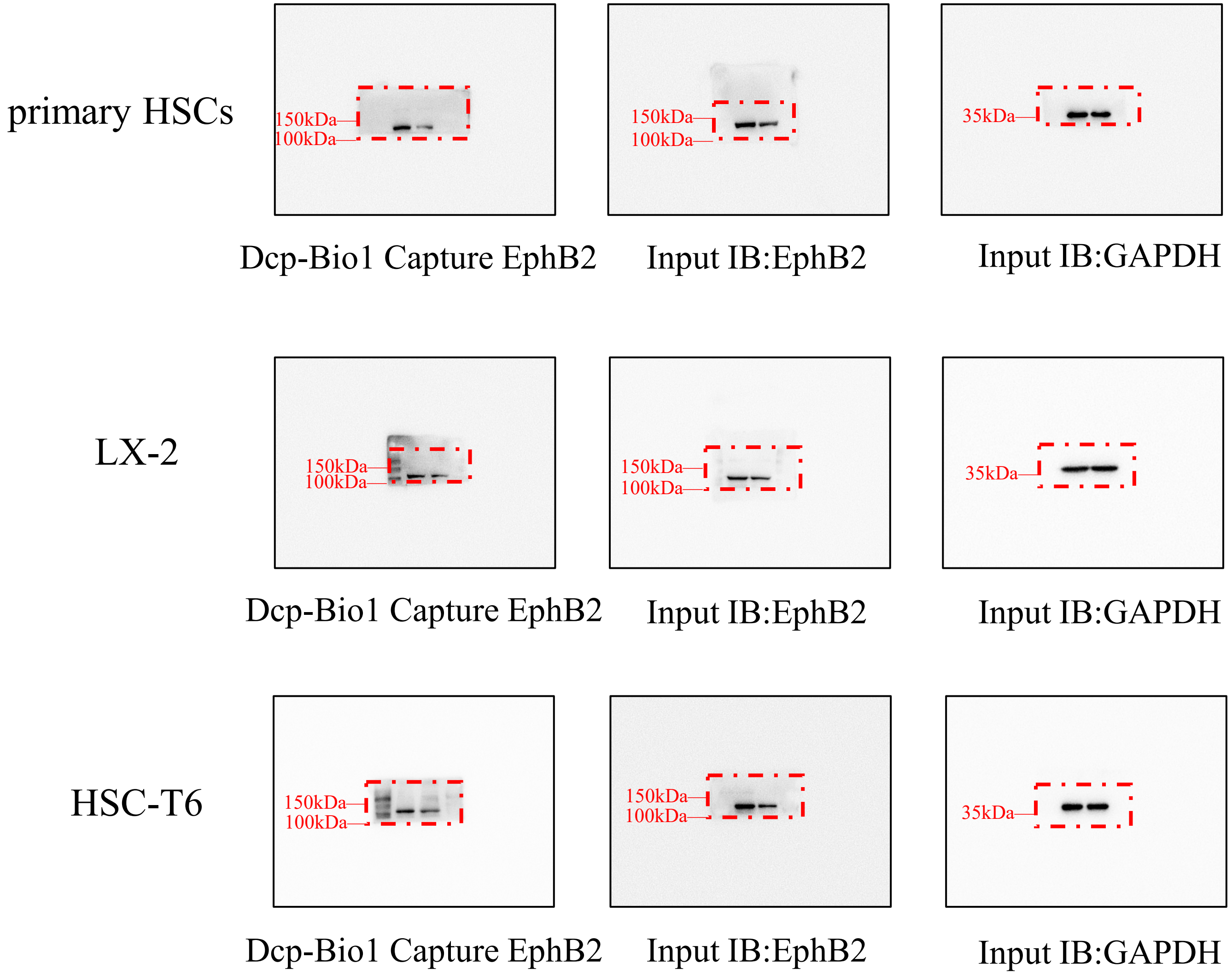
primary HSCs
150kDa
100kDa
35kDa
Dcp-Bio1 Capture EphB2
Input IB:EphB2
Input IB:GAPDH
LX-2
150kDa
100kDa
35kDa
Dcp-Bio1 Capture EphB2
Input IB:EphB2
Input IB:GAPDH
HSC-T6
150kDa
100kDa
35kDa
Dcp-Bio1 Capture EphB2
Input IB:EphB2
Input IB:GAPDH

Fig 2A

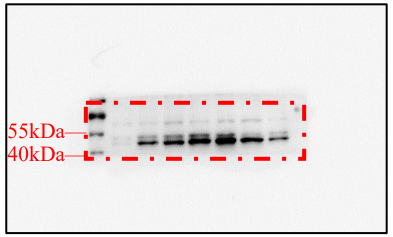

Dcp-Bio1 Capture EphB2 (His)

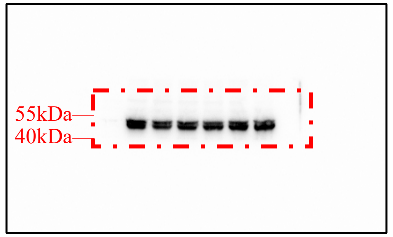

Input:EphB2 (His)

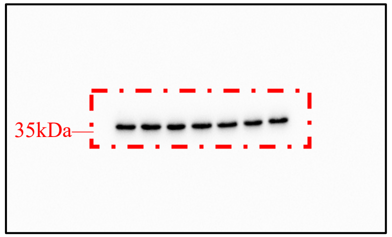

Input IB:GAPDH

Fig 2B

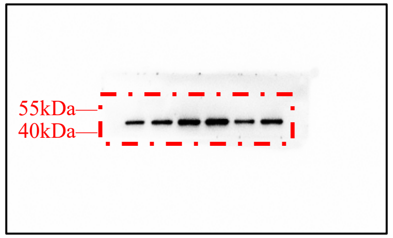

Dcp-Bio1 Capture EphB2 (His)

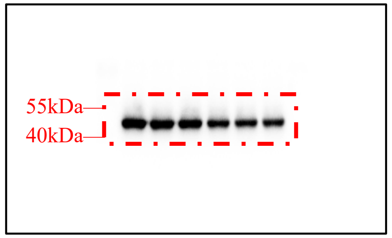

Input:EphB2 (His)

Fig 2E

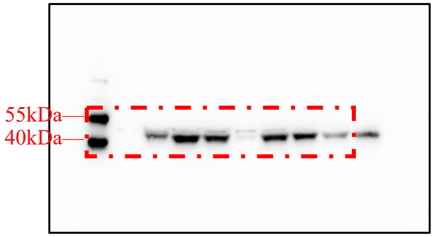

Dcp-Bio1 Capture EphB2 (His)

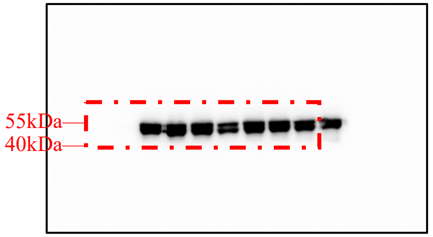

Input:EphB2 (His)

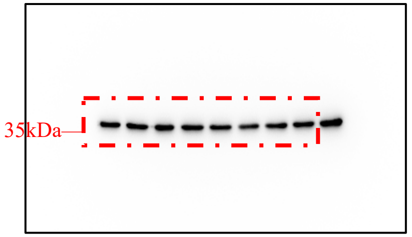

Input IB:GAPDH

Fig 3A

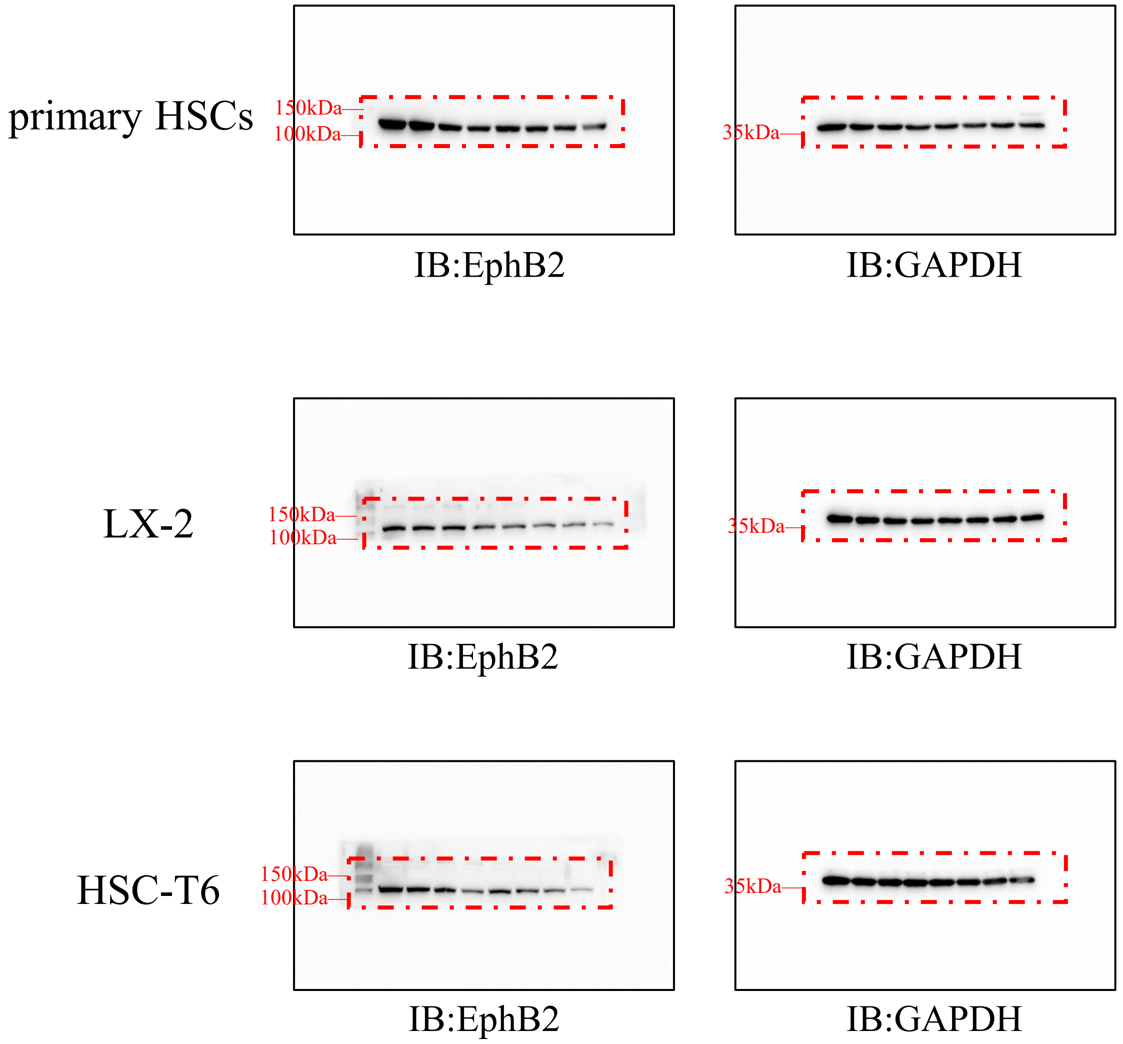
primary HSCs
150kDa
100kDa
35kDa
IB:EphB2
IB:GAPDH
LX-2
150kDa
100kDa
35kDa
IB:EphB2
IB:GAPDH
HSC-T6
150kDa
100kDa
35kDa
IB:EphB2
IB:GAPDH

Fig 3B

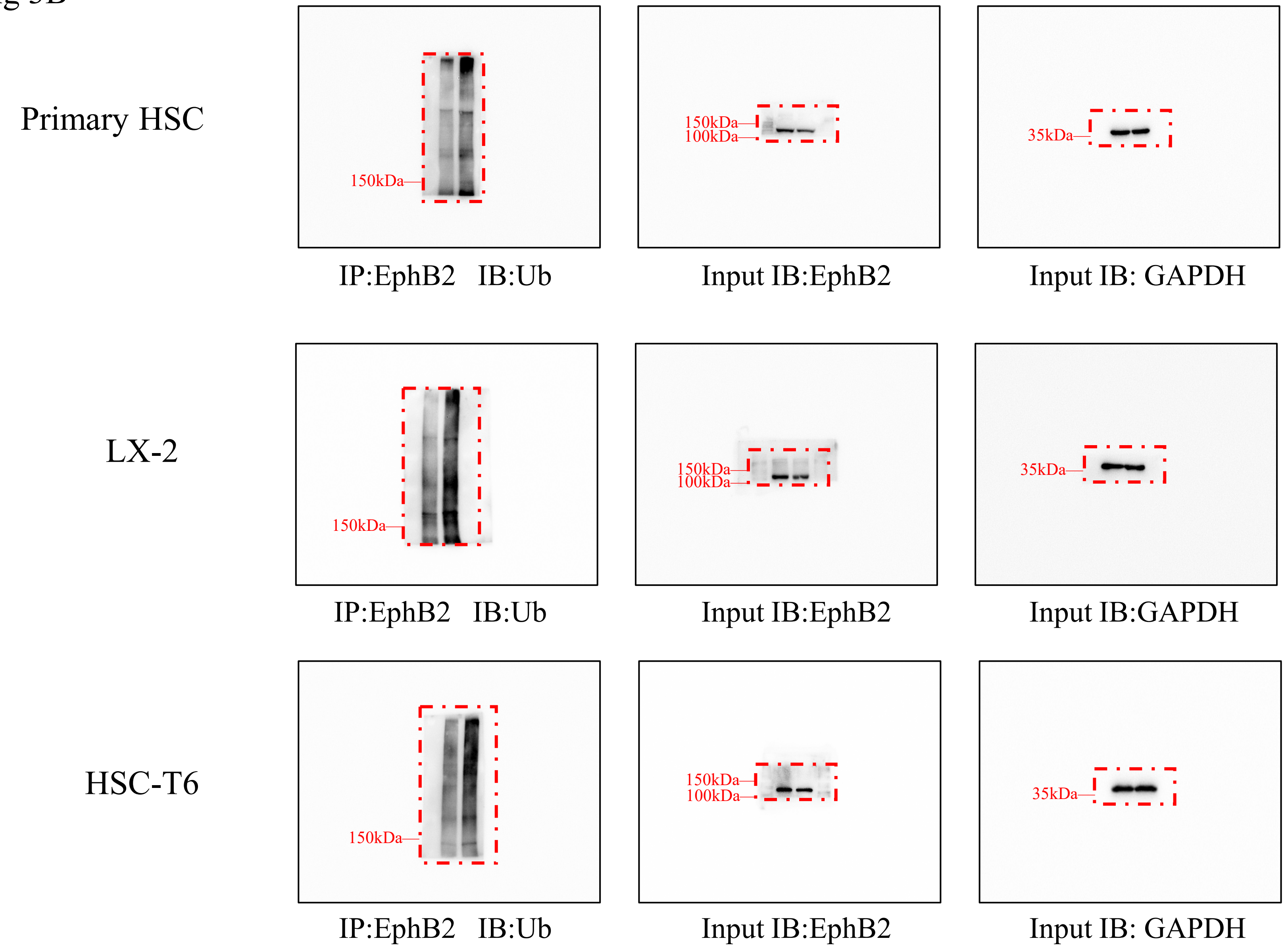
Primary HSC
150kDa
IP:EphB2 IB:Ub
150kDa
100kDa
Input IB:EphB2
35kDa
Input IB: GAPDH
LX-2
150kDa
IP:EphB2 IB:Ub
150kDa
100kDa
Input IB:EphB2
35kDa
Input IB:GAPDH
HSC-T6
150kDa
IP:EphB2 IB:Ub
150kDa
100kDa
Input IB:EphB2
35kDa
Input IB: GAPDH

Fig 3C

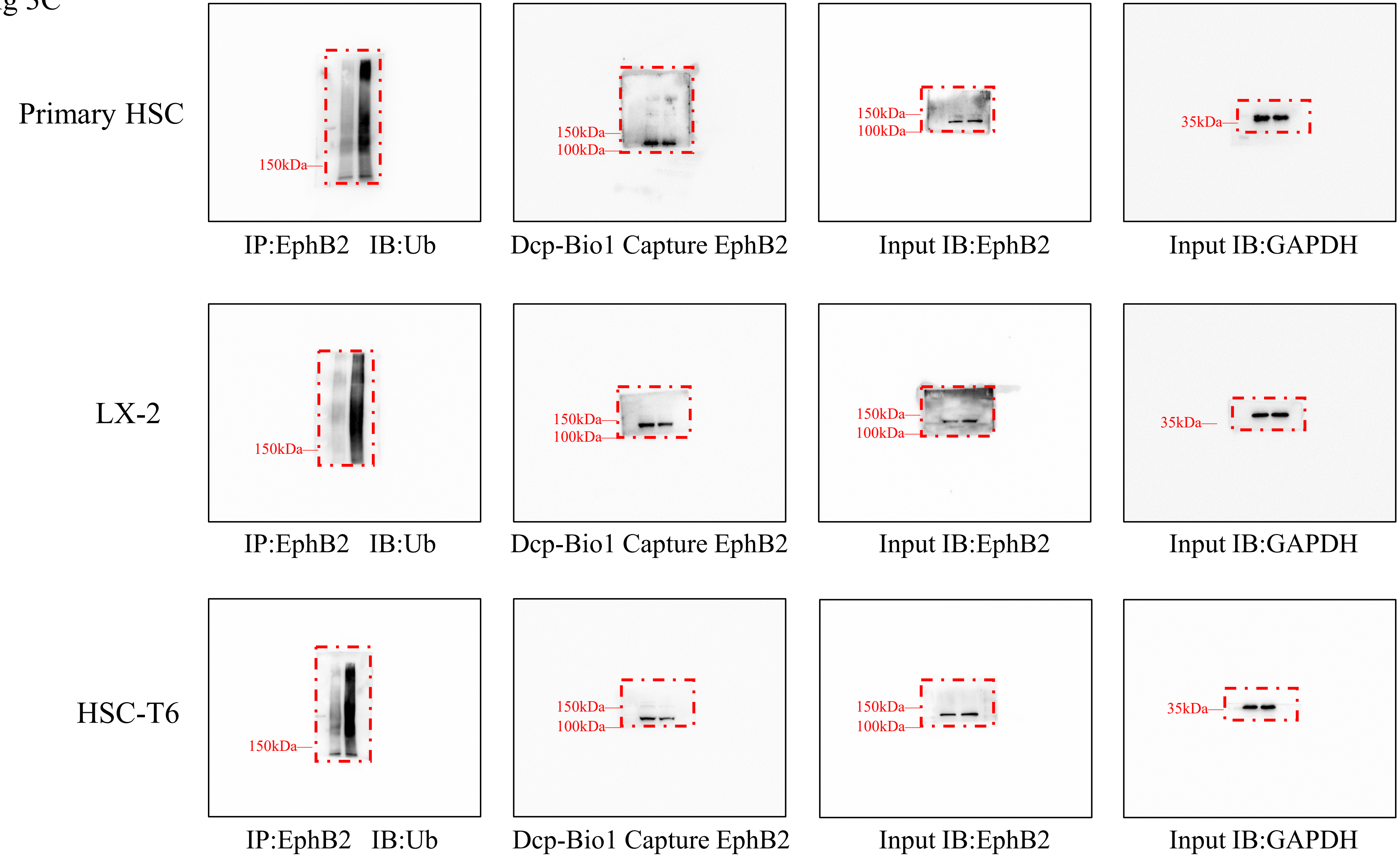

Primary HSC
LX-2
HSC-T6
150kDa
100kDa
35kDa
IP:EphB2 IB:Ub
Dcp-Bio1 Capture EphB2
Input IB:EphB2
Input IB:GAPDH

Fig 3D

LX-2-EphB2-KO

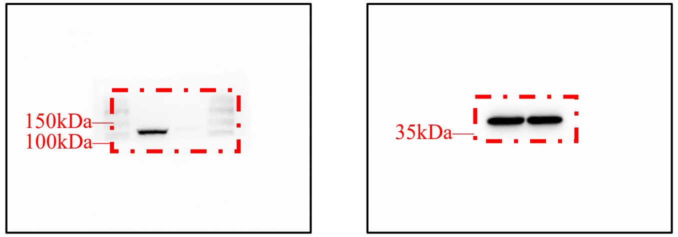

IB:EphB2 IB:GAPDH

Fig 3E

HEK293T

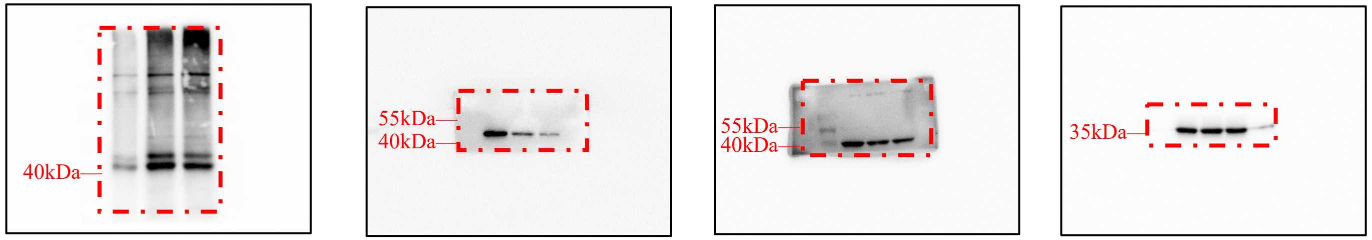

IP:His IB:Ub Dcp-Bio1 Capture EphB2 Input IB:His Input IB:GAPDH

Fig 3F

HEK293T

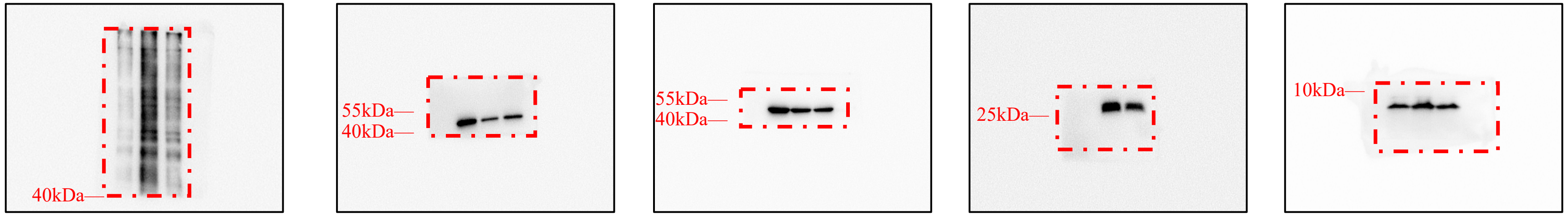

IP:His IB:HA Dcp-Bio1 Capture EphB2 Input IB:His Input IB:Myc Input IB:HA

Fig 4D

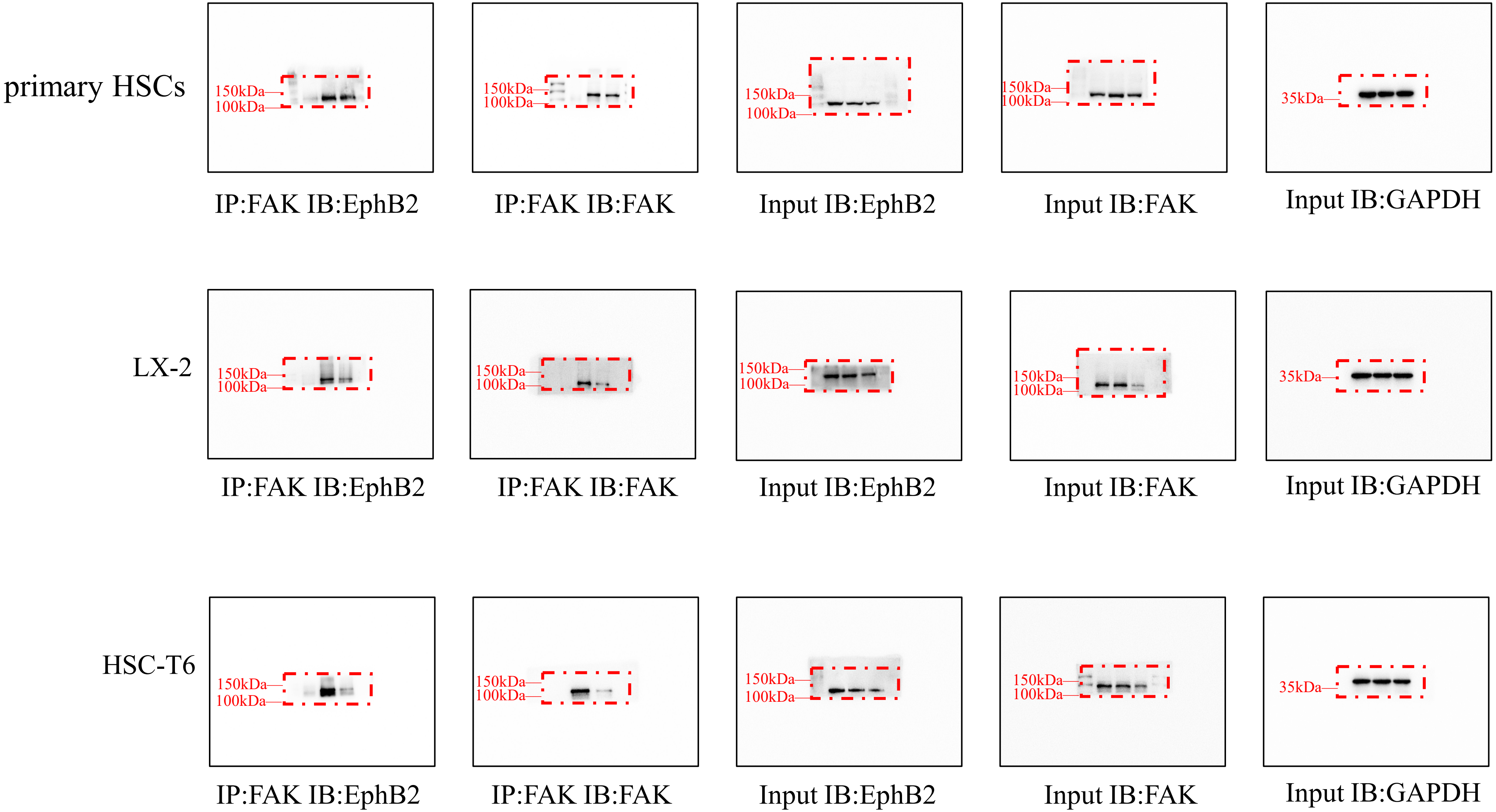
primary HSCs
LX-2
HSC-T6
150kDa
100kDa
35kDa
IP:FAK IB:EphB2
IP:FAK IB:FAK
Input IB:EphB2
Input IB:FAK
Input IB:GAPDH

Fig 4F

HEK293T

HEK293T

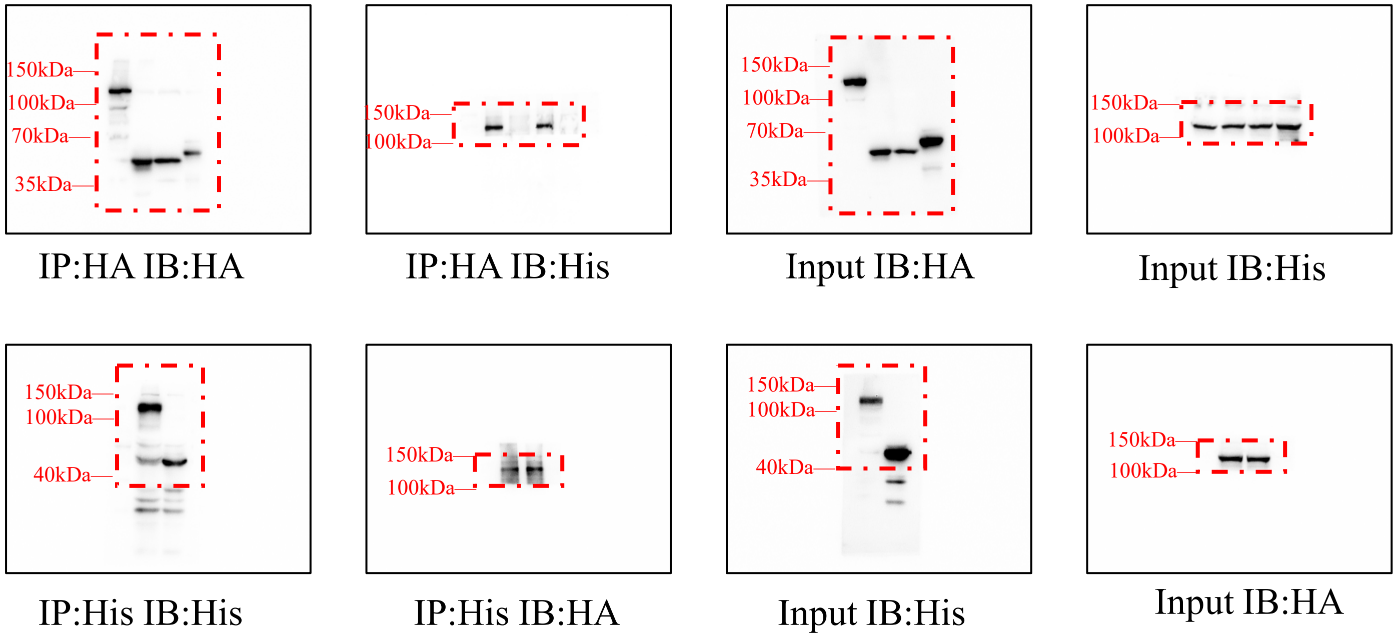

Fig 4G

LX-2-EphB2-KO

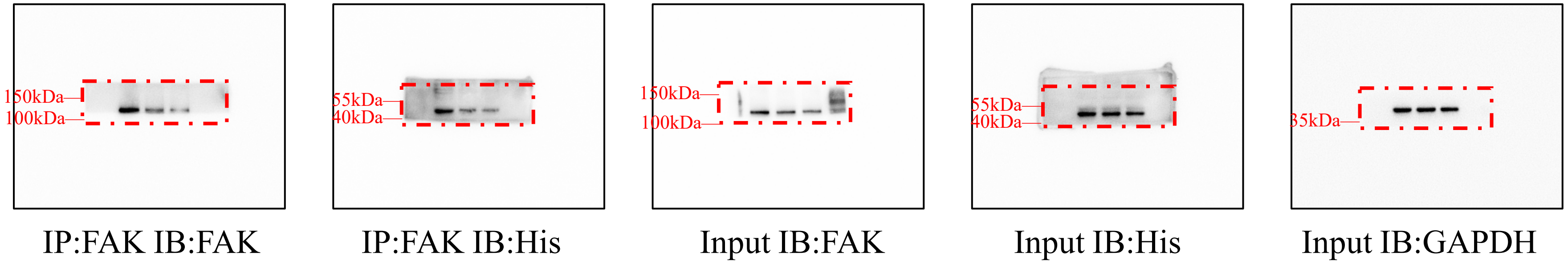

Fig 4H

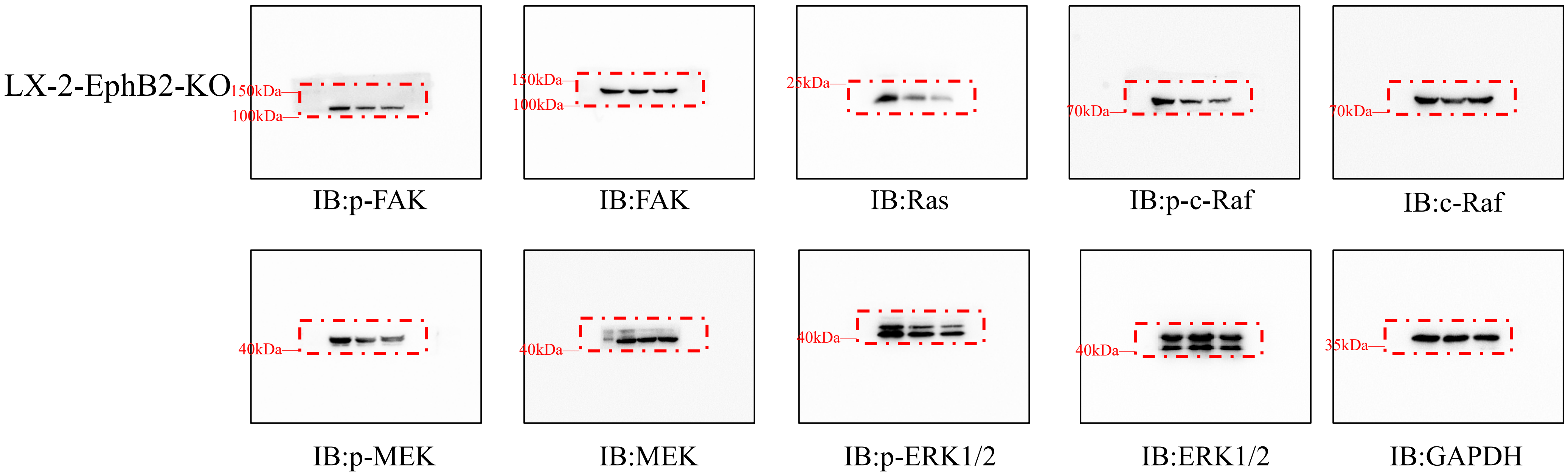

LX-2-EphB2-KO
150kDa
100kDa
IB:p-FAK
150kDa
100kDa
IB:FAK
25kDa
IB:Ras
70kDa
IB:p-c-Raf
70kDa
IB:c-Raf
40kDa
IB:p-MEK
40kDa
IB:MEK
40kDa
IB:p-ERK1/2
40kDa
IB:ERK1/2
35kDa
IB:GAPDH

Fig 5E

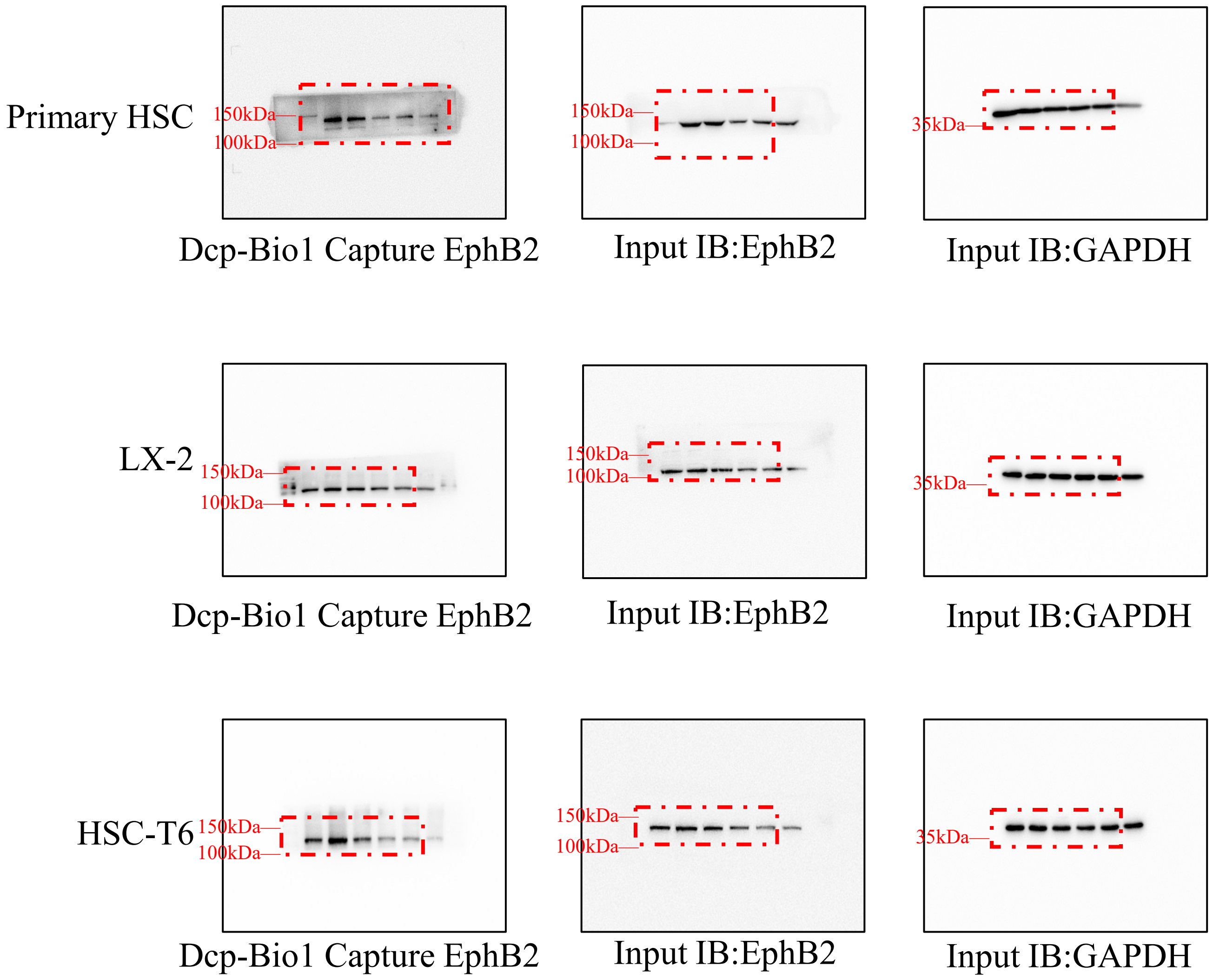
Primary HSC
150kDa
100kDa
Dcp-Bio1 Capture EphB2
150kDa
100kDa
Input IB:EphB2
35kDa
Input IB:GAPDH
LX-2
150kDa
100kDa
Dcp-Bio1 Capture EphB2
150kDa
100kDa
Input IB:EphB2
35kDa
Input IB:GAPDH
HSC-T6
150kDa
100kDa
Dcp-Bio1 Capture EphB2
150kDa
100kDa
Input IB:EphB2
35kDa
Input IB:GAPDH

Fig 5F

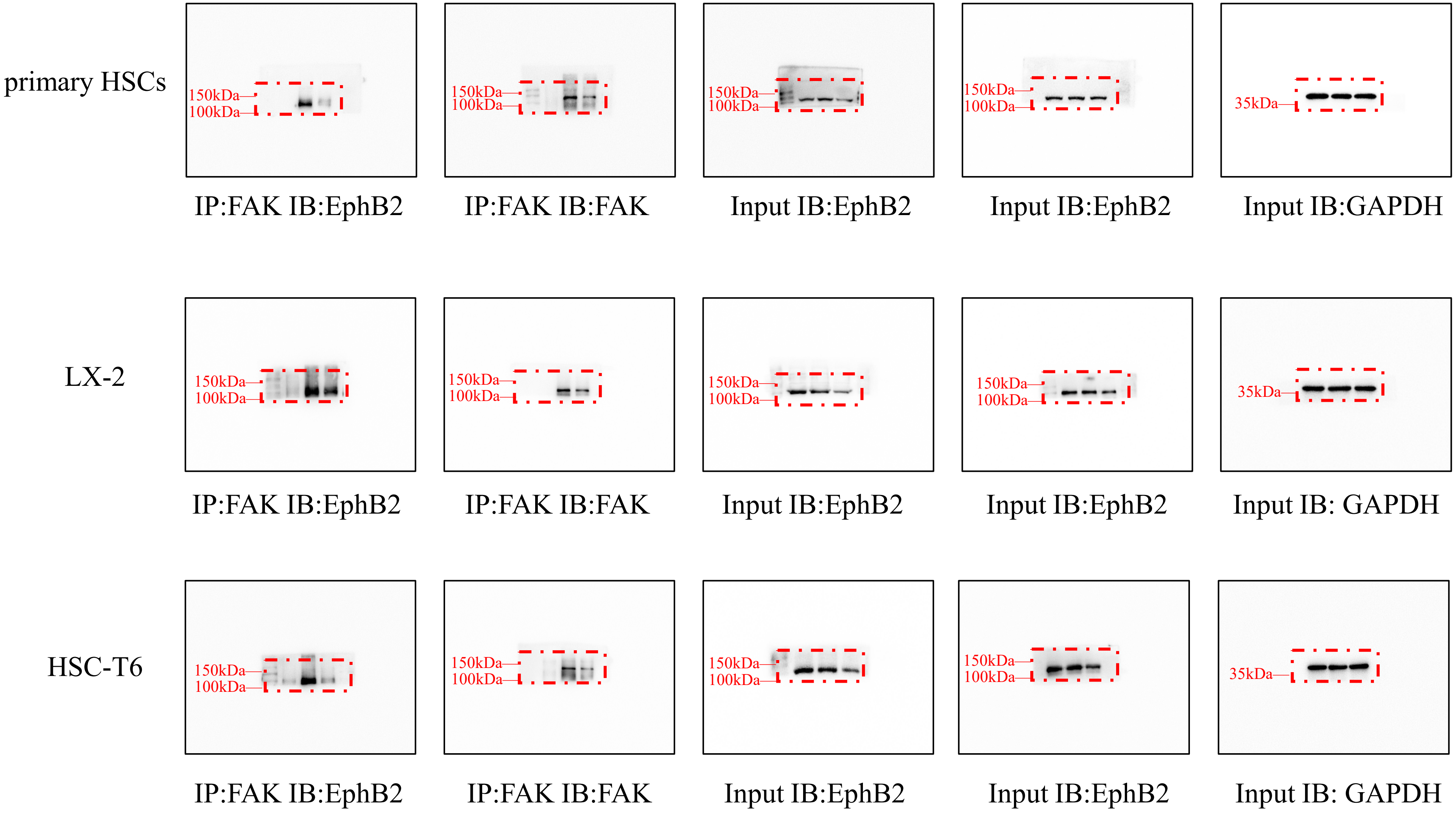
primary HSCs
150kDa
100kDa
IP:FAK IB:EphB2
150kDa
100kDa
IP:FAK IB:FAK
150kDa
100kDa
Input IB:EphB2
150kDa
100kDa
Input IB:EphB2
35kDa
Input IB:GAPDH
LX-2
150kDa
100kDa
IP:FAK IB:EphB2
150kDa
100kDa
IP:FAK IB:FAK
150kDa
100kDa
Input IB:EphB2
150kDa
100kDa
Input IB:EphB2
35kDa
Input IB: GAPDH
HSC-T6
150kDa
100kDa
IP:FAK IB:EphB2
150kDa
100kDa
IP:FAK IB:FAK
150kDa
100kDa
Input IB:EphB2
150kDa
100kDa
Input IB:EphB2
35kDa
Input IB: GAPDH

Fig 5G

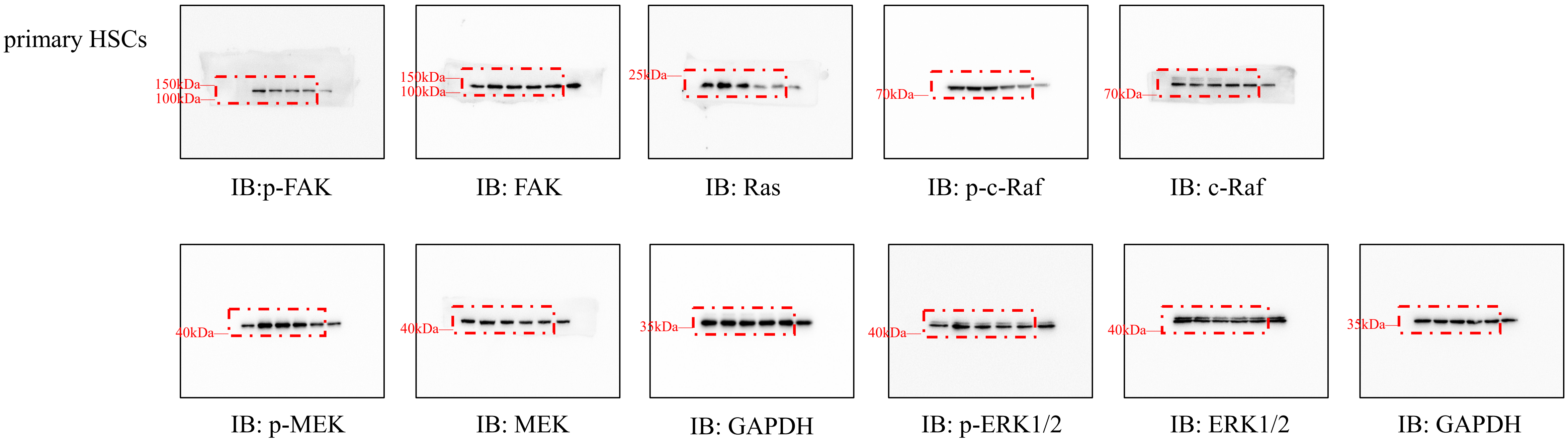
primary HSCs
150kDa
100kDa
IB:p-FAK
150kDa
100kDa
IB: FAK
25kDa
IB: Ras
70kDa
IB: p-c-Raf
70kDa
IB: c-Raf
40kDa
IB: p-MEK
40kDa
IB: MEK
35kDa
IB: GAPDH
40kDa
IB: p-ERK1/2
40kDa
IB: ERK1/2
35kDa
IB: GAPDH

Fig 5G

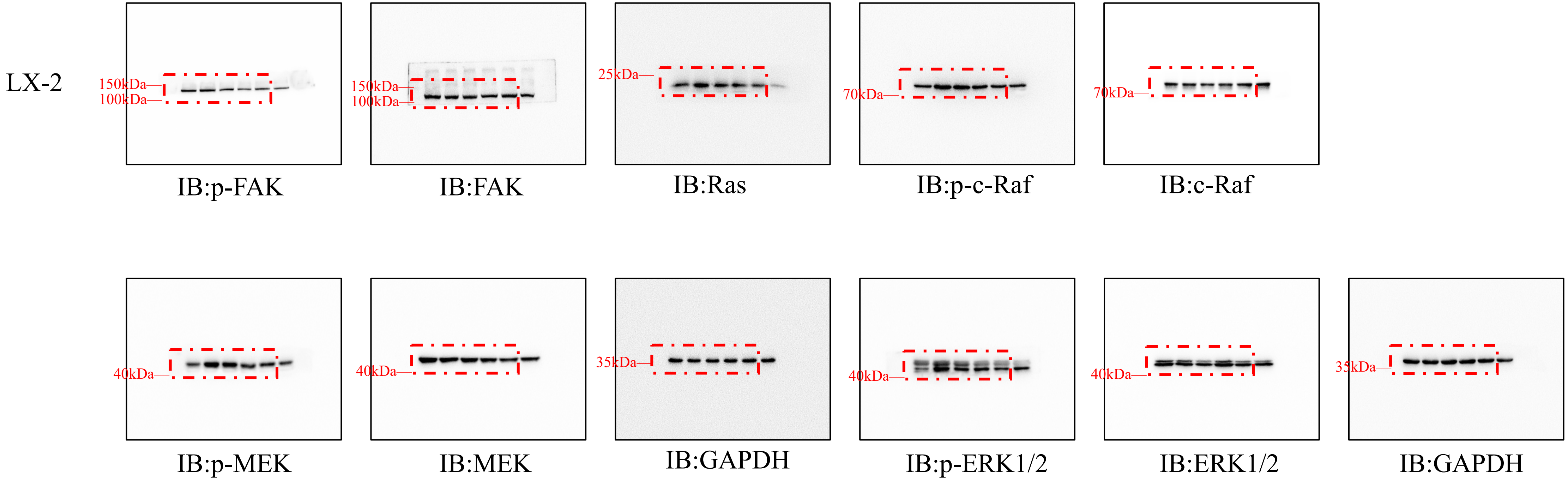
LX-2
150kDa
100kDa
IB:p-FAK
150kDa
100kDa
IB:FAK
25kDa
IB:Ras
70kDa
IB:p-c-Raf
70kDa
IB:c-Raf
40kDa
IB:p-MEK
40kDa
IB:MEK
35kDa
IB:GAPDH
40kDa
IB:p-ERK1/2
40kDa
IB:ERK1/2
35kDa
IB:GAPDH

Fig 5G

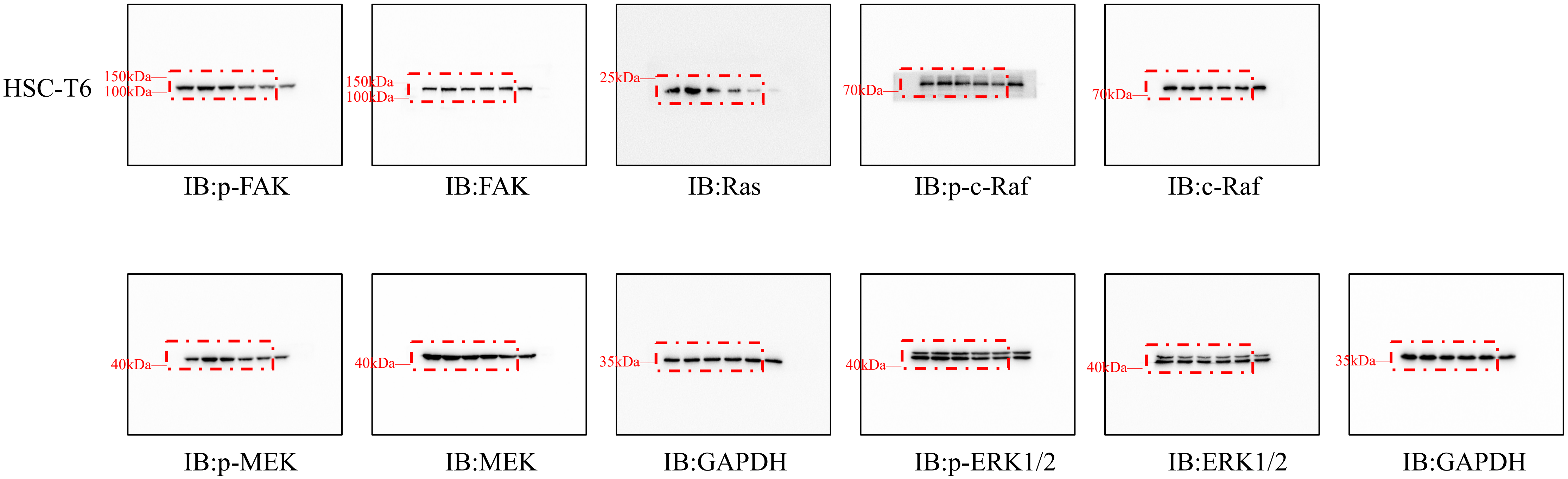
HSC-T6
150kDa
100kDa
IB:p-FAK
150kDa
100kDa
IB:FAK
25kDa
IB:Ras
70kDa
IB:p-c-Raf
70kDa
IB:c-Raf
40kDa
IB:p-MEK
40kDa
IB:MEK
35kDa
IB:GAPDH
40kDa
IB:p-ERK1/2
40kDa
IB:ERK1/2
35kDa
IB:GAPDH

Fig S4C

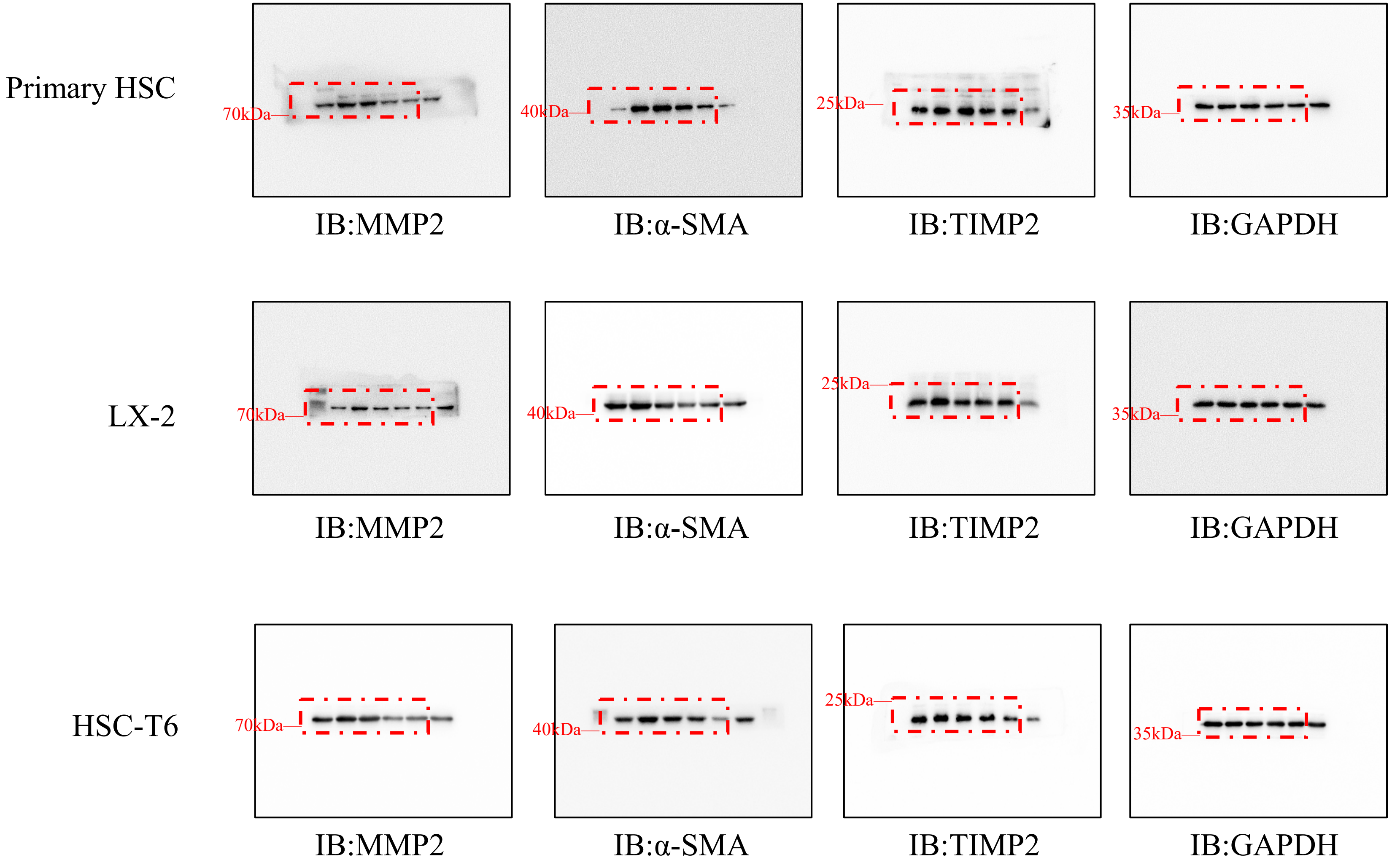
Primary HSC
70kDa
IB:MMP2
40kDa
IB:α-SMA
25kDa
IB:TIMP2
35kDa
IB:GAPDH
LX-2
70kDa
IB:MMP2
40kDa
IB:α-SMA
25kDa
IB:TIMP2
35kDa
IB:GAPDH
HSC-T6
70kDa
IB:MMP2
40kDa
IB:α-SMA
25kDa
IB:TIMP2
35kDa
IB:GAPDH

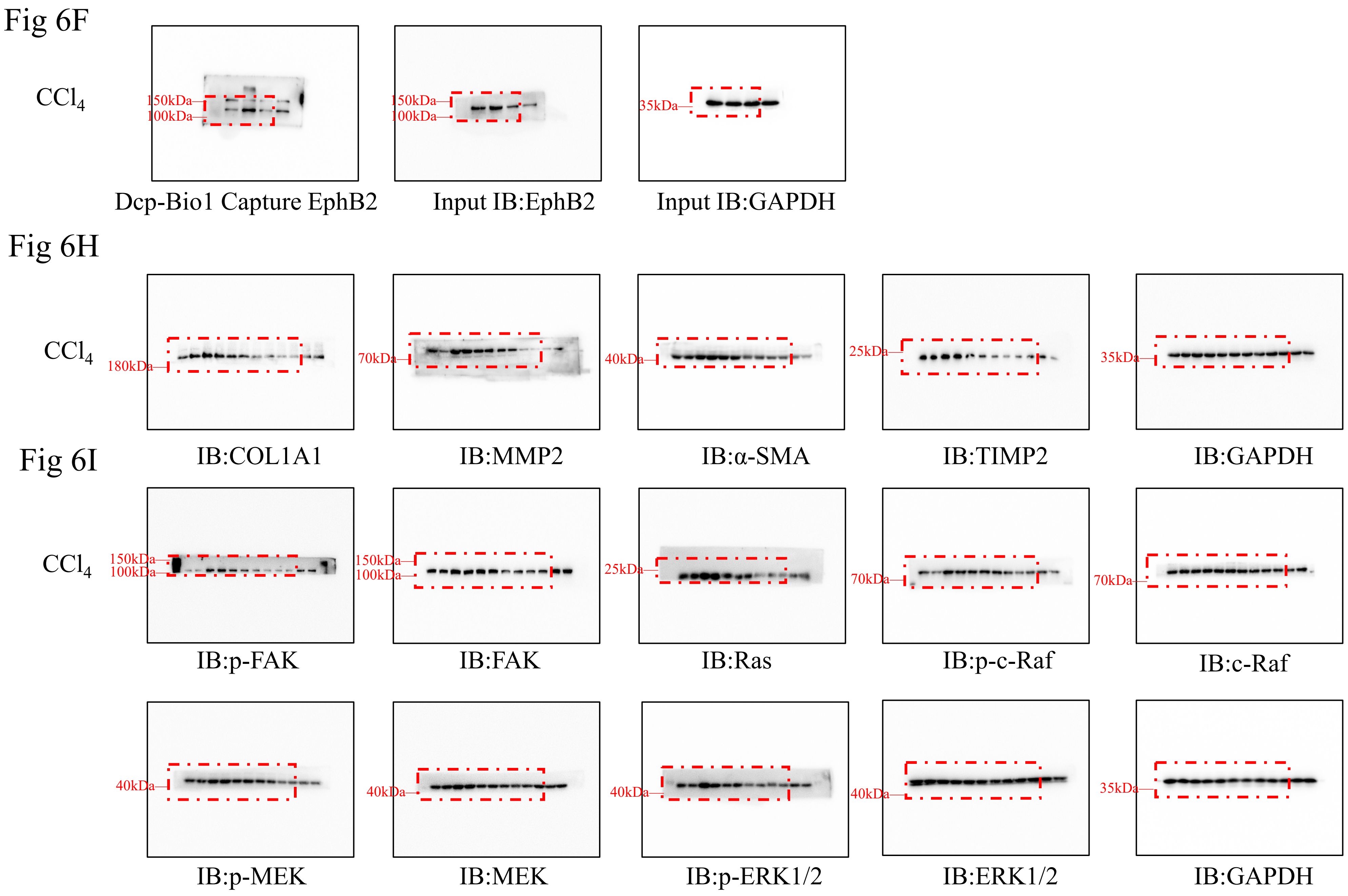
Fig 6F
CCl4
150kDa
100kDa
Dcp-Bio1 Capture EphB2
150kDa
100kDa
Input IB:EphB2
35kDa
Input IB:GAPDH
Fig 6H
CCl4
180kDa
IB:COL1A1
70kDa
IB:MMP2
40kDa
IB:α-SMA
25kDa
IB:TIMP2
35kDa
IB:GAPDH
Fig 6I
CCl4
150kDa
100kDa
IB:p-FAK
150kDa
100kDa
IB:FAK
25kDa
IB:Ras
70kDa
IB:p-c-Raf
70kDa
IB:c-Raf
40kDa
IB:p-MEK
40kDa
IB:MEK
40kDa
IB:p-ERK1/2
40kDa
IB:ERK1/2
35kDa
IB:GAPDH

Fig 7F

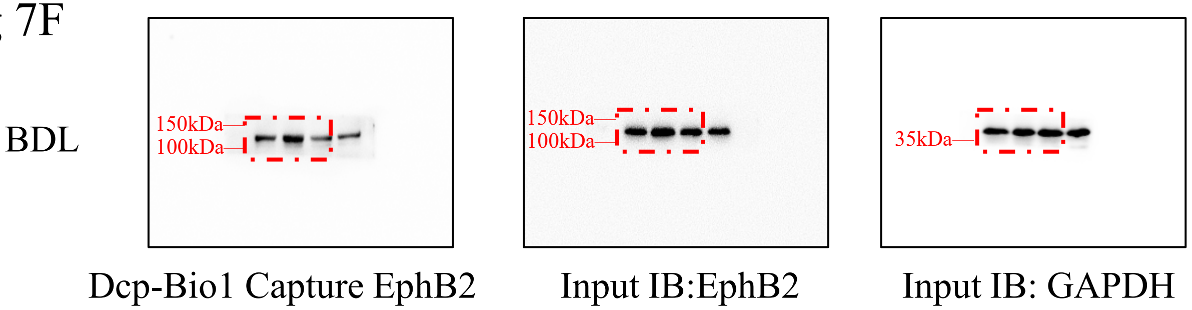

Fig 7H

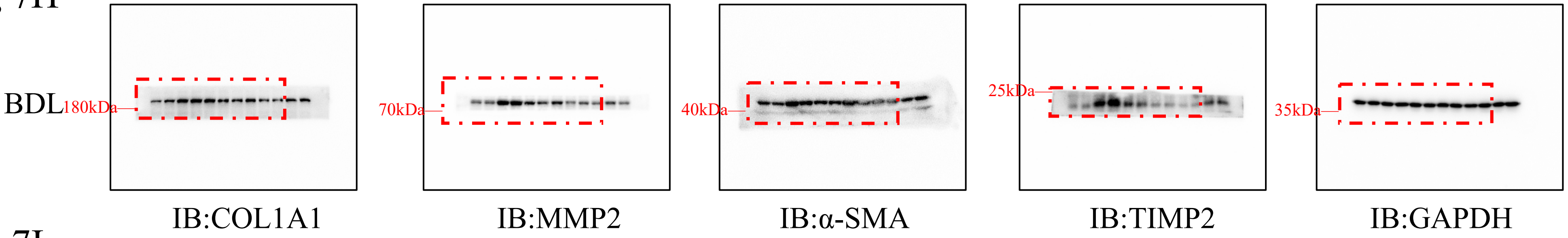

Fig 7I

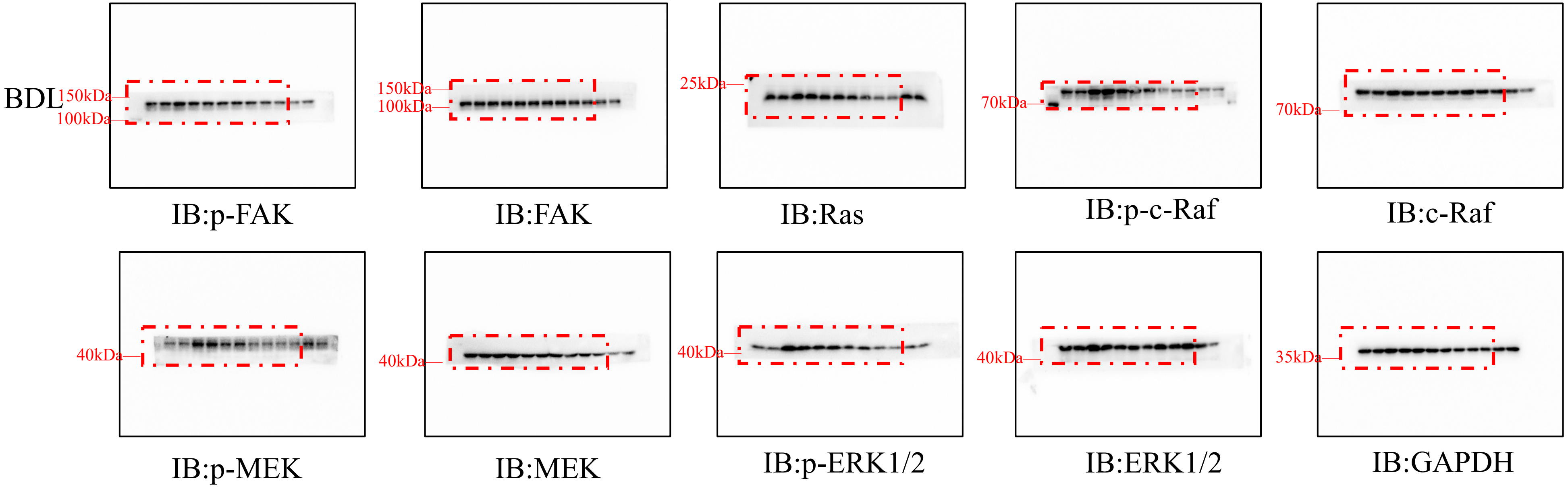

Supplement: Supplementary file 2 — Original Western Blots [file 41419_2024_6997_MOESM2_ESM.pdf]
